# Recent Advances in Periodontal Regenerative Medicine: A Focus on the Role of Mechanical Stimulation

**DOI:** 10.3390/biomedicines13112839

**Published:** 2025-11-20

**Authors:** Lidiia Grinchevskaia, Daria Revokatova, Mohammad Hadi Norahan, Alexey Senkovenko, Frederico David Alencar de Sena Pereira, Nastasia Kosheleva, Anastasia Shpichka, Peter Timashev

**Affiliations:** Institute for Regenerative Medicine, I.M. Sechenov First Moscow State Medical University, 119991 Moscow, Russianorakhan_m_kh@staff.sechenov.ru (M.H.N.);

**Keywords:** periodontal regeneration, mechanobiology, periodontal ligament stem cells, mechanical stimulation, biomaterials

## Abstract

Periodontitis is a prevalent chronic inflammatory disease that leads to the progressive destruction of periodontal tissues and remains the primary cause of tooth loss worldwide. Despite advances in regenerative approaches—including stem cell therapy, scaffold-based tissue engineering, and guided tissue regeneration—the complete and functional restoration of the periodontal ligament remains a major clinical challenge. Stem-cell-based therapies and advanced biomaterials have emerged as promising strategies in regenerative medicine, offering potential for restoring periodontal structure and function. Among cells, periodontal-ligament-derived stem cells (PDLSCs) show exceptional regenerative potential due to their ability to differentiate into cementoblasts, osteoblasts, and other cell types essential for periodontal repair. In recent years, a variety of biomaterials with distinct specifications and properties have been utilized to repair periodontal damage. In addition to the inherent properties of biomaterials, the morphology and structural characteristics of these materials as bioequivalents for periodontal regeneration are also critical considerations. Furthermore, recent studies emphasize that mechanical stimulation plays a considerable role in directing stem cell differentiation, gene expression, matrix organization, and modulating inflammatory responses in periodontal regeneration. Canonical parameter ranges for systematic analysis indicate that cyclic stretch strain of 1–20% at 0.1–0.5 Hz (6–30 cycles/min) typically increases the expression of osteogenic markers (RUNX2, ALP, OCN) and matrix components (Col1) in PDLSCs. Conversely, higher values (>15%) often bias the response toward inflammatory pathways (IL-6, PGE2). Static compression above 2 g/cm^2^ consistently stimulates the secretion of pro-inflammatory cytokines (IL-6, IL-8) and alters the RANKL/OPG balance in favor of osteoclastogenesis. Significant heterogeneity in response across studies will be analyzed by examining key methodological variables, including specific loading regimens (duration, frequency patterns) and culture conditions (e.g., serum/osteogenic supplements), which critically modulate mechanotransduction outcomes. This review summarizes current progress in periodontal regenerative medicine, emphasizing cellular and biomaterial considerations, as well as biofabrication techniques, with a particular focus on the influence of mechanical forces on PDLSCs. We discuss cellular responses to mechanical stimuli, including changes in gene expression, cytoskeletal organization, proliferation, and differentiation. Combining biological knowledge with advances in bioprinting and the study of mechanobiology, we finally discuss promising opportunities for improving periodontal regeneration that can be applied in the future in clinical practice.

## 1. Introduction

Periodontal disease (PD), or periodontitis, is one of the world’s most common oral diseases, with a global prevalence of up to 50%; approximately 1.1 billion individuals (13.1% of the global population) suffer from severe forms of the disease [1]. PD is a chronic inflammatory condition affecting the periodontium, namely structures such as the gingiva, periodontal ligament, cementum, and alveolar bone (AB) [2]. Characterized by clinical attachment loss, alveolar bone resorption, periodontal pocketing, and gingival inflammation, periodontitis is the leading cause of tooth loss and decreased quality of life [3]. The periodontal ligament (PDL) is an essential component of the periodontium, providing physical support, nutrition, and development for teeth. Therefore, the health of the PDL significantly affects the outcome of periodontal disease. Studies have shown that removing PDL cells from the cementum can lead to ankylosis and root resorption. However, a regenerated PDL, with properly organized fibers integrated into the newly formed cementum, can help prevent epithelial tissue from moving downward and retain precursor cells needed for bone and cementum regeneration, ultimately leading to better clinical outcomes [4]. Despite extensive research aiming to regenerate periodontal structures, effective treatments remain a major challenge in clinical practice. Current therapeutic approaches, including nonsurgical treatments and guided tissue regeneration (GTR), often fail to achieve comprehensive regeneration of the periodontal apparatus, particularly the PDL, which is key to preserving tooth function. The chronic inflammation associated with periodontitis complicates repair efforts, emphasizing the need for innovative therapies that can effectively reconstruct functional PDL.

The goal of effective periodontal therapy is to completely restore all components of the periodontium to their normal structure and function, consisting of the gingival connective tissue, cementum, AB, and PDL [5]. Stem cell therapy has emerged as a promising tool in medicine and tissue engineering due to its regenerative capabilities and immunomodulatory properties, offering potential solutions for enhancing periodontal regeneration and improving clinical outcomes in patients with periodontitis. Most studies focus on the application of various types of stem cells for periodontal therapy, including odontogenic cells such as dental pulp stem cells (DPSCs), periodontal-ligament-derived stem cells (PDLSCs), gingival stem cells (GSCs), and mesenchymal stem cells (MSCs) from different sources such as bone marrow, adipose tissue, and umbilical cord.

Among these cell types, PDLSCs appear to be the most suitable for the regeneration of the periodontal complex itself, primarily due to their ability to differentiate into cementoblasts, fibroblasts, and osteoblasts [6]. GSCs represent a promising alternative. Their key advantages include high accessibility for harvesting and significant anti-inflammatory potential, which is crucial for treating the inflammatory environment of periodontitis [6,7,8]. However, there are some contradictory results about GSCs, which will be described in more detail below. DPSCs possess a high proliferative capacity but are less effective than PDLSCs and GSCs in specifically regenerating the functional periodontal structures [6]. Among MSCs, BM-MSCs remain a powerful tool with proven efficacy in bone regeneration. However, their clinical application is often limited by the highly invasive nature of the harvesting procedure [6]; thus, the application of adipose tissue and UC-MSCs is a promising alternative. Adipose-tissue-derived mesenchymal stem cells (AT-MSCs) also have advantages, as they have a high anti-inflammatory potential [9], are effective in the treatment of periodontitis [10], and, importantly, can be obtained through a simple and non-invasive procedure. Moreover, utilization of advanced scaffold materials, essential in tissue engineering and regenerative medicine, is vital for addressing the shortcomings of conventional periodontal therapies. These materials are designed to repair and replace damaged tissues to restore physiological function [5,11] and were selected for their low toxicity and compatibility, attributed to their distinctive chemical properties. Mechanically, they must possess sufficient strength and resilience to maintain integrity and facilitate tissue growth. Moreover, these biomaterials, specifically designed for periodontal regeneration, should enhance PDL regeneration and be osteoconductive; osteoinductivity refers to the ability to stimulate progenitor cells to differentiate into osteoblasts, which are responsible for bone formation [12]. Despite considerable progress in the translation of regeneration techniques for gingiva and AB, the structural and functional characteristics of a regenerated PDL remain unreplicated by these therapies. Consequently, tissue engineering represents a novel approach to addressing these complex issues [13].

Researchers are exploring the enhancement of tissue regeneration and maturation by employing various signals to the cells embedded in engineered constructs, alongside the utilization of proficient biomaterials for tissue engineering [14]. The periodontal ligament requires mechanical stress for optimal function [15]. Mechanical forces and stimulation have been regarded as significant external signals for the regeneration of PDL tissue [15]. It has been shown that applying various mechanical forces to periodontal ligament cells promotes changes in gene expression responsible for cell differentiation [16,17], the balance of pro- and anti-inflammatory cytokines and growth factors [18,19], as well as reorganization of the cytoskeleton, cell morphology [20], and cell death [17]. These processes create conditions that closely mimic natural physiological stimuli within the body, enabling the optimization of bioequivalents and tissue constructs for effective regeneration of the periodontal complex.

The environment of periodontal biomechanics is extremely complicated. When mechanical loading is applied, the cementum–periodontal ligament (PDL)–alveolar bone complex must be seen as a whole because each of its parts has its own mechanical properties [21,22]. However, the biomechanical qualities of the periodontal ligament (PDL) are extremely important because it functions as a damper by absorbing the masticatory forces from the high loads applied by the teeth and their periodontal supporting tissues during mastication [23]. Due to the tooth’s intricate geometry, the periodontal ligament experiences varying forms of strain based on where it is located beneath the tooth. When the tooth is exposed to external loading, the apexes experience the highest compressive strain, and the side of the root experiences the highest tensile strain. It has been demonstrated that the kind of loading that collagen bundles can withstand is connected with their morphological characteristics and orientation. For this reason, it is critical to comprehend how the PDL behaves under different loading scenarios [23]. The biomechanical properties of PDLs are fundamentally anisotropic, determined by the specific orientation of principal collagen fiber bundles (e.g., oblique, horizontal, apical) that are arranged in a way that directs and supports loads. The PDL is a viscoelastic and poroelastic medium that relaxes stress, creeps, and has properties that change with frequency. When dynamic compressive loading is applied within physiological ranges (e.g., 0.05 Hz to 5 Hz), its storage and loss moduli increase. The tissue experiences low-magnitude strains, and its mechanical properties (stiffness, compression modulus, and viscoelasticity) vary. For example, the stiff, supportive collar area is different from the furcation zone, which is more flexible. This complicated relationship between fiber alignment, time-dependent damping, and regional heterogeneity makes sure that the PDL can send orthodontic forces through the tooth–bone complex without hurting it. This allows for a quantitative analysis of the mechanobiological responses that follow [24,25].

In this review paper, we primarily highlight periodontal regeneration, specifically focusing on periodontal ligament regeneration within the context of tissue engineering, including aspects of cellular biology, biomaterial considerations, and biofabrication methods. Next, we explore more extensively how mechanical stimulation affects periodontal ligament stem cells, specifically examining the role of mechanical forces on the underlying mechanisms that influence various cellular functions, such as gene expression, proliferation, and differentiation.

In the third periodontal complex, a variety of mechanical effects occur, which can be classified into four main types of cells: position (extension), extension (compression), and shear (shear). Each of these types of forces has substantive significance: cyclic structures and elongation predominate during chewing and orthodontic tooth movement; abrupt loads in the marginal periodontal areas. For a systematic analysis, this review will consider the effects in several quantitative groups: cyclic change of 1–20% strain at a frequency of 6–30 cyc/min, static elongation of 2–25 g/cm^2^, and shear loads of 0.5–10 dynes/cm^2^. These ranges partially correspond to the conditions of chewing loads, as well as orthodontic effects, with particularly high values often simulating pathological effects, which ensures the relevance of the analyzed experimental data in vivo in the conditions of the periodontal complex.

To ensure a comprehensive and reproducible synthesis of the current evidence, a systematic literature search was conducted. The electronic databases PubMed/MEDLINE, Scopus, Google Scholar, and Web of Science were searched for articles without strict restrictions on publication years; however, the focus was primarily on more recent articles published since 2015 (some earlier articles that appeared interesting to the authors for analysis were also included in this review). The search strategy utilized a combination of keywords and MeSH terms related to (“periodontal regeneration” OR “periodontal engineering” OR “PDL regeneration” OR “periodontal complex regeneration” OR “periodontal complex” OR “periodontal cells”) AND (“mechanical stimulation” OR “mechanotherapy” OR “cyclic strain” OR “fluid shear stress” OR “stretching” OR “compression” OR “mechanical forces” OR “stretch bioreactor” OR “compression bioreactor” OR “shear stress bioreactor”). The initial search results were screened by title and abstract to identify relevant fundamental research, preclinical (in vitro and in vivo), and key clinical studies. Articles were included if they mainly looked at how certain mechanical forces affect periodontal ligament cells, alveolar bone, or cementum in the context of regeneration. We also looked at articles about the structure and cellular composition of the periodontal complex, biomaterials, and biofabrication strategies. After that, the full texts of the chosen articles were found and read to obtain information on the type of mechanical stimulation, the biological outcomes, and the suggested molecular mechanisms. These were then put together in a narrative form to meet the review’s goals.

## 2. Periodontal Ligaments

**Figure 1:**
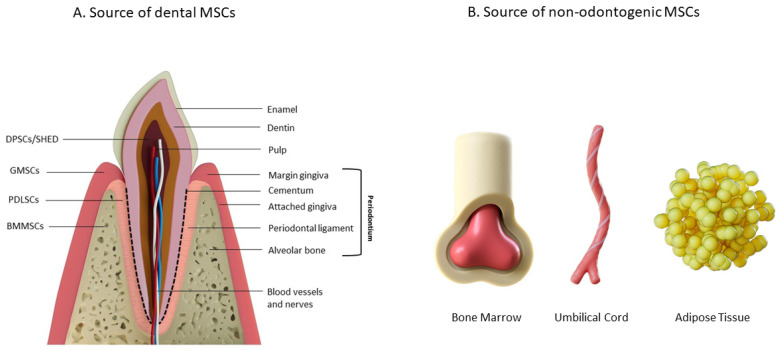
Structure of periodontal complex and sources of MSCs for periodontitis therapy. (**A**) Periodontal complex and source dental MSCs; (**B**) sources of non-odontogenic MSCs.

The periodontium is a complex structure that is essential for providing mechanical stability and anchoring the teeth within the alveolar sockets. The periodontium comprises the gingival complex, AB, periodontal ligament, and root cementum [26,27]. The gingiva, in turn, is subdivided into marginal (unattached) gingiva, which represents the terminal edge of the gingiva surrounding the tooth, and attached gingiva, which is a continuation of the marginal gingiva and is connected to the periosteum of the AB [28]. In the gingiva complex, there are also the gingival margin and the gingival sulcus. The first is the gum’s visible edge, and the second is a shallow groove around the tooth, bounded by the tooth surface and the gingival margin. This groove is an important diagnostic criterion for disturbances in the periodontal complex. Normally, its depth should range from 0.5 to 3 mm; however, in cases of periodontal issues, it can significantly increase, forming so-called periodontal pockets [27,28]. Root cementum is a densely calcified connective tissue lacking a vascular network. It covers the roots of teeth and is necessary for attaching the main fibers of the periodontal ligament. There are two main types of cementum: cellular extrinsic fiber cementum and cellular intrinsic fiber cementum. The first is located on the cervical half or two-thirds of the root. It is termed thus because its formative cells remain on its surface [26,27]. The degree of mineralization in this area varies between 45 and 60% [26]. The second type of cementum is characteristic for areas along the apical third or half of the root and in furcation regions. This type of cementum is less mineralized compared to the cellular extrinsic fiber cementum. For the cellular intrinsic fiber cementum, two cell types are most characteristic: cementoblasts (which form collagen fibers) and cementocytes (cells “trapped” in lacunae) [26,27]. AB is part of the jaw where the roots of teeth are located. It forms during tooth eruption and participates in anchoring the developing periodontal ligament to bone [26]. Each alveolus is surrounded on both sides by outer cortical plates, which are supported by Haversian systems. The spongy (trabecular) bone occupies an intermediate position between these cortical plates. At the same time, the bone lining directly around the alveolar socket is called the bundle bone because it provides attachments for bundles of periodontal ligament fibers [27]. Additionally, these regions contain a large number of principal fibers of the periodontal ligament—so-called Sharpey’s fibers. The periodontal ligament (PDL) is a soft connective tissue that is located between the hard tissues—the root of the tooth and the AB [15,24]. The width of the PDL ranges from 0.15 to 0.38 mm [24]. The main components of PDL are loose connective tissue, fibers, and cells [29]. The main component of this fibrous structure (PDL’s extracellular matrix (ECM)) is collagen, especially type I. These subsequently create fibrils, which then interlace to form bundles of fibers. In the PDL space, the fibers are oriented mainly radially but gradually assume a circular arrangement near the cement and AB, ensuring the attachment of PDL to the latter [24]. The cellular composition of PDL includes fibroblasts, osteoblasts, osteoclasts, cementoblasts, odontoclasts, epithelial, stem, and immune cells [24,30]. Fibroblasts are the major cells of this composition, which withstand high dynamic loads and mechanical forces, as well as synthesize and digest fibrillar collagen with high intensity. The half-life of collagen fibers in PDL is only a few days, and this process is provided by PDL fibroblasts: they are able to both remove damaged collagen fibers and form new ones, and fibroblasts need mechanical action to remodel the matrix [30]. Scientists are particularly interested in the stem cells found in the periodontal ligament—PDLSCs [29]. Self-renewing PDLSCs can differentiate into osteoblasts, cementoblasts, fibroblasts, and osteoclasts [24]. Collagen fibers rapidly renew, ensuring the integrity of the PDL tissue. Moreover, the fibers linked to bone and cement experience ongoing remodeling due to the heightened activity of osteoclasts, osteoblasts, and cementoblasts, whose population renewal is facilitated by PDLSCs [30]. The detailed structure of the periodontal complex is shown in Figure 1.

The periodontium’s unique anatomical architecture serves as a highly specialized mechanotransduction system rather than a static scaffold [24]. The complex structure and composition of the PDL influence its biomechanical behavior, closely interacting with the surrounding biomechanical environment [23]. The collagenous network is hierarchically organized, ranging from the nanoscale to the mesoscale, where tropocollagen molecules aggregate into fibrils, which subsequently arrange into collagen fibers, bundles, and fascicles. Every hierarchical level improves the overall mechanics and function of the periodontal ligament (PDL), thus facilitating the effective delivery of forces from the tooth to the alveolar bone. It is well known that the organization and composition of the collagen network play a critical role in the mechanical characteristics of the periodontal ligament (PDL) [31]. In order to convert the complex occlusal loads on the tooth into controlled tensile strains along their axes, the periodontal ligament’s (PDL) fibers are oriented in a particular oblique manner [31]. This direct physical connection sends mechanical energy straight to the embedded periodontal ligament fibroblasts and stem cells. These cells are closely linked to the collagen network through integrin-mediated focal adhesions. Also, the high turnover rate and short half-life of periodontal collagen are not just metabolic oddities; they make an extracellular matrix that is always ready to be remodeled in response to mechanical stimuli [32]. This allows for more rapid architectural changes than would be possible with a more stable connective tissue. In vitro modeling of this system necessitates the consideration of in vivo boundary conditions: the primary mechanical stimulus for periodontal ligament (PDL) cells is cyclic tensile strain, and culture models should strive to replicate this particular loading mode instead of depending exclusively on static or compressive stimuli to provoke physiologically pertinent responses [24].

## 3. Cellular Considerations

Periodontitis is a progressive deterioration of the structures that support teeth, such as periodontal ligaments, AB, gingiva, and cementum, which can ultimately lead to tooth loss. Stem cell therapies in periodontal tissue regeneration reported optimistic results and advantages over traditional methods and were demonstrated in lots of clinical trials, especially for gingiva and AB tissue reconstruction. An analysis of fifteen studies that examined the effect of stem cell therapies on periodontal tissue regeneration demonstrated that cell therapy significantly improves attachment level and stimulates bone mineralization and gingiva reconstruction [7].

In periodontitis treatment, traditional methods such as guided tissue regeneration (GTR) have been widely applied to restore the health of the periodontal ligament. This surgical procedure involves the placement of membranes that cover the bone and periodontal ligament and temporarily separate them from the gum epithelium and connective tissue, creating a physical barrier to prevent the proliferation of epithelial cells into the periodontal pocket, thereby maintaining a small space for tissue regeneration [33]. However, this approach alone is insufficient to predict the regeneration of periodontal tissues and should be supplemented with other regenerative methods, such as bone grafting. At the same time, the use of bone grafts is limited to the regeneration of AB and does not encompass the complete regeneration of all periodontal tissues [23].

The main advantage of cell therapy is its comprehensive approach and the ability to restore all components of the periodontal complex simultaneously. This is largely due to the multi-differentiation potential of the cells used in the therapy.

Most models of periodontal disease involve disorders affecting all components of the periodontium. Therefore, the studies described below will primarily focus on integrated therapeutic approaches and rarely concentrate solely on the regeneration of the periodontal ligament. However, review will emphasize the effects that therapy has on the periodontal ligament.

Generally, there are two types of cells that can be used for periodontal tissue therapy: odontogenic cells, such as DPSCs or SHED (stem cells from human exfoliated deciduous teeth), PDLSCs, gingiva stem cells (GSCs), stem cells from the apical papilla (SCAPs), AB marrow–derived MSCs (BMMSCs) and non-odontogenic mesenchymal stem cells (MSCs). These come from different sources, such as bone marrow, adipose tissue, and umbilical cord. All cell sources are demonstrated in Figure 1.

### 3.1. Odontogenic Cells

Odontogenic cells are often referred to as dental stem cells (DSCs) and they share similar traits with other MSCs, such as self-renewal and multipotency [34].

Dental pulp stem cells (DPSCs) or SHEDs (stem cells from human exfoliated deciduous teeth) were described in 2000 [34] as the first type of identified DSCs. They assist in oral tissue repair and regeneration functions and can be easily obtained in large numbers because they can be obtained from extracted human teeth [35]. DPSCs are predecessors for odontoblasts which are responsible for dentin repair. Due to their neural origin, DPSCs can undergo neuronal differentiation, secrete factors to protect neurons [36], and stimulate angiogenesis.

DPSCs’ ability to generate dentin/pulp-like complexes was demonstrated more than 20 years ago: after a subcutaneous co-transplantation with hydroxyapatite/tricalcium phosphate (HAp/TCP) matrices, researchers observed the formation of vascularized pulp tissue surrounded by a well-defined layer of odontoblast-like cells, aligned around mineralized dentin [37].

Also, DPSC-derived exosomes promote the osteogenesis of PDLSCs and decrease inflammation [38].

DPSCs are widely used in the treatment of bones [39,40,41], but based on recent analyses, they are less effective [42] for periodontal tissue regeneration than PDLSCs. For therapeutic applications, DPSCs can be utilized individually, as suspensions, aggregates, cell sheets [43], or with matrices [44]; alternatively, their secretions, such as exosomes and chemokines, can be employed [38].

The effectiveness of DPSC suspension and cell sheets was demonstrated in a pig model of periodontitis caused by a surgical AB defect. Histology and computed tomography (CT) analyses were used to demonstrate the regeneration of bone and the formation of a cementum-like layer [43]. In a recent article, the advantage of using cell sheets in comparison with cell suspension was shown on periodontal bone defects in pigs. Grafting stem cells as sheets led to a higher amount of new bone at 12 weeks (*p* < 0.01) compared to the grafting of dissociated cells [43,45]. In another study, the efficacy of DPSCs on the Bio-Oss^®^ (Geistlich Pharma, Wolhusen, Switzerland) scaffold was demonstrated in a dog periodontitis model [44].

Clinical trials have also been conducted. A quasi-experimental study with 22 patients between 55 and 64 years of age with PD demonstrated that treatment with DPSCs on the collagen scaffold showed an increase in bone mineral density. An analysis of saliva samples revealed increased superoxide dismutase and decreased levels of IL1β, which highlighted the potential antioxidant and anti-inflammatory effects of this therapy [46].

Periodontal ligament-derived stem cells (PDLSCs) are classified as MSCs and they express cell surface markers that are similar to those of MSCs derived from bone marrow. According to recent data, PDLSCs differ from MSCs in several parameters [47], such as high expression of the tendon-associated marker scleraxis and their ability to form Sharpey’s fibers and cementum [48]. Since they are located in the perivascular wall of periodontal ligaments, they share similarities with pericytes in morphology, cell phenotype, and differentiation potential [49]. Due to their ability to differentiate in cementoblasts, odontoblasts, and osteoblasts, PDLSCs are considered to be the best type of stem cell for periodontal tissue regeneration, and have been shown to be even more effective than DPSCs [42].

Despite sharing common characteristics, both PDLSCs and GSCs exhibit significant donor-to-donor heterogeneity [50]. Single-cell RNA sequencing analyses of PDLSCs and GSCs reveal significant but limited differences in gene expression, pathway enrichment, and protein–protein interaction (PPI) networks between donors. These are significant, but they are also limited. This highlights the importance of considering donor-specific transcriptomic variability before clinical application. Furthermore, donor age is a critical factor influencing stem cell function [51,52]. With increasing age, PDLSCs demonstrate a marked decline in regenerative capacity, which is characterized by reduced proliferative and differentiation potential, increased rates of apoptosis, and diminished immunomodulatory abilities [52]. These age-related impairments are linked to the accumulation of cellular damage, including telomere shortening [51]. Therefore, both donor-specific variability and donor age should be considered as crucial selection criteria for developing effective periodontal tissue regeneration therapies.

Furthermore, PDLSCs are heterogeneous and consist of distinct subpopulations. For instance, ALP+ cells have been shown to exhibit elevated levels of the stemness markers STRO-1 and CD146, along with higher expression of the pluripotency genes NANOG, OCT4, and SOX2 in early passages [53]. PDLSCs also can differ from each other in osteogenic potential. For instance, studies have shown that cells expressing the Zbp1 gene exhibit significant potential for osteogenic differentiation; this potential can be changed via genome editing or viral vectors [47].

More than 20 years ago, it was proved that PDLSCs have the potential to be used in creating periodontal ligament complexes, since they are able to differentiate into functional cementoblasts and form collagen fibers embedded in cementum-like tissue after subcutaneous injection in mice [54]. Since then, PDLSCs have repeatedly been used for periodontal complex therapy applied alone or with other cells or matrices.

It was demonstrated that PDLSC injection promotes periodontal regeneration in a mice model of periodontitis caused by the removal of AB. Two or four weeks after cell injection, higher values of new bone width, new bone area fraction, and cementum-like tissue were observed [55]. In another study, PDLSCs were applied in the treatment of miniature pig periodontitis caused by surgical AB defects. Twelve weeks after transplantation, cell suspension and cell sheets caused bone repair, which was proved by histological and CT analysis. The cells stimulated the formation of cementum-like layers and b-globin expression. Interestingly, human DPSCs sheets demonstrated a greater effect than suspension [43]. Iwasaki’s group conducted a novel study in which human PDLSCs were transferred onto decellularized amniotic membranes to serve as a scaffold and then were transplanted into periodontal defects in rats. Their findings revealed that this method significantly enhances periodontal healing compared to the transplantation of amnion alone. The researchers assessed the formation of cementum, periodontal ligament, and bone using micro-CT and histological analysis [56].

PDLSCs for therapy also can be applied in combination with other cells. For example, composite cell sheets from rat PDLSCs and mice-osteoblast-like cells were found to promote mice periodontitis treatment through the formation of PDL-like fibers and AB periodontal ligament structures; this finding was determined based on immunohistochemical (IHC) analyses for the expression of periostin and osteocalcin (OCN) and azan staining for PDL-like fibers [57]. The same results were obtained in rat models when human PDLSCs were used. Besides their effectiveness being evidenced in animal models, the benefits of PDLSCs have been widely demonstrated in lots of clinical trials [7].

In addition to the ability of PDLSCs to differentiate into components of the periodontal complex, their regenerative potential is also supported by two important features: firstly, the regulation of the oral microbiome by increasing the abundance of non-pathogenic bacteria and inhibiting the growth of pathogenic bacteria [55]; secondly, they participate in immune reactions. For example, PDLSCs have the ability to inhibit the proliferation of T and B lymphocytes, as well as stimulate the induction of M2 anti-inflammatory polarization in macrophages [29]. In earlier research studies, cells isolated from the periodontal ligament were referred to as periodontal ligament fibroblasts (PDLFs) [58,59]. Later, the terminology became more general, and these cells were called periodontal ligament cells (PDLCs) [60]. In recent scientific articles, a different term has been adopted—periodontal ligament stem cells (PDLSCs) [17]. All the above-mentioned names are still found in the literature today. However, in this study, the periodontal ligament cells are referred to as PDLSCs.

Gingiva stem cells (GSCs) are a distinctive homogenous subset of MSCs which develop from neural ectomesenchyme along with contributions from the perifollicular mesenchyme and the dental follicle proper. Compared to other types of DSCs, they have great accessibility and incredible long culture sustainability [61]. These cells have been repeatedly used to treat diseases such as chronic inflammatory diseases like colitis, rheumatoid arthritis, systemic lupus erythematosus, and diabetes [62]. However, results for their use in the treatment of periodontitis are conflicting. There are works where a positive effect has been shown in the treatment of periodontitis. For example, in combination with fibroin/chitosan oligosaccharide lactate hydrogel, these cells were used in a rat model to treat periodontitis caused by cotton ligatures. Histology and micro-CT analyses revealed reduction in inflammation and destruction. But the application hydrogel only also had a good impact, so maybe we over-estimate the cell’s role [63]. Another study demonstrates that hyaluronic acid (HA) hydrogel with GSCs can be effective in the treatment of periodontitis and significantly improved probing depth, clinical attachment level (CAL), and gingival recession (GR) [64]. In another study, the application of STRO-1-positive GSCs onto a collagen scaffold and deproteinized bovine cancellous bone was used in the treatment of periodontal defects in miniature pigs. Cell-loaded scaffolds showed superior improvements such as the regeneration of the bone, cementum, and periodontal ligament [64]. The same results were obtained when GSCs sheets were used in treating dog periodontitis, caused by class III furcation defects (5 mm from the furcation fornix to the bottom of the defect) with the addition of anaerobic bacteria into the defect [65].

On the contrary, another study showed that, among PDLSCs, AT-MSCs, and gingival margin cells, GSCs have the least regenerative potential [66] and Vaquette et al. demonstrated negative effects of GSC sheets compared to using the scaffold without the cells: decreased bone volume/total volume (BV/TV), cementum formation, and ligament fibers attachment [67]. According to the authors, the difference between these studies can be explained by cellular heterogeneity. The findings suggest that the application of primary, unsorted GSC cultures—comprising both progenitor cells and terminally differentiated fibroblasts—is ineffective for periodontal regeneration unless the population is first enriched for stem cells through sorting [68]. Furthermore, the notable inconsistencies in regenerative outcomes—such as the negative effects of GSCs on bone volume (BV/TV) and cementum formation in a sheep model [67] versus their positive effects in rodent models [63]—underscore the significant influence of the chosen animal model on experimental results.

### 3.2. Mesenchymal Stem Cells (MSCs)

MSCs-based therapy is a novel and promising approach for periodontal tissue regeneration. Their effectiveness is due not only to their self-renewal and differentiation potential, but also to the release of extracellular vesicles, which promote periodontal regeneration by modulating inflammation and regulation of function of osteoclasts [69]. Among MSCs, cells from bone marrow, adipose tissue, and umbilical cord are the most commonly used for periodontal tissue therapy. Although there is not yet an MSCs product approved in the clinic for PD treatment, the preclinical and clinical studies showed that they could significantly improve periodontal regeneration [70].

Bone marrow MSCs (BM-MSCs) are widely used for clinical application purposes in regenerative medicine and are especially useful for treating osteogenesis, but this approach is imperfect because of its differentiation potential. According to lots of preclinical studies [71], BM-MSCs also can ameliorate periodontitis through the regeneration PDL fibers, cementum, and AB [67,72,73]. BM-MSCs can be applied as a suspension [72] or can be modified with bone morphogenic protein (BMP-7) [73] or pretreated with acetylsalicylic acid (ASA), which has been reported to be beneficial for MSCs use in terms of inflammation control and tissue regeneration. In ligature- and bacteria-induced periodontitis, a model with rats showed that ASA-BM-MSCs treatment reduced inflammatory infiltration and AB loss; this was proved by immunohistochemistry staining of osteoprotegerin (OPG) with a receptor activator of nuclear factor kappa ligand (RANK-L) and micro-CT [74]. Also, BM-MSCs can be used with scaffolds or with hydrogels [67]. BM-MSCs, applied on an biphasic electrospinning scaffold, demonstrated high efficacy in a sheep periodontitis model that led to cementum formation and ligament fibers attachment after 10 weeks of therapy [67]. The main disadvantage of BM-MSCs is the rather traumatic and invasive method used to isolate them from the patient. So, MSCs from adipose tissue (AT-MSCs) or umbilical cords (UC-MSCs) are more convenient to use due to the simplicity of the method used to obtain them.

Adipose tissue MSCs (AT-MSCs) have recently been a focus of attention in regenerative medicine due to their high regenerative potential and the relatively easy and non-invasive method used for obtaining them, making them suitable for personalized medicine. They exhibit impressive self-renewal capabilities, rapid proliferation, have notable anti-inflammatory effects, and support tissue remodeling and angiogenesis [75]. The positive effect of AT-MSCs during periodontitis therapy can be attributed to some different factors. First, it was shown that AT-MSCs exhibit periodontal ligament differentiation capacities (expression of Asporin, Periostin, Col2, Lumican) [76]. They are also known to have a high anti-inflammatory potential since they have high expression of the immune suppressive factors GBP4 and IL1-RA upon treatment with an inflammatory cytokine cocktail of interferon gamma (IFN-γ), tumor necrosis factor-α (TNF-α), and IL-6 [76]. Also, AT-MSCs exosomes were demonstrated to stimulate periodontal regeneration [9,77].

AT-MSCs application in cell suspension showed excellent result in a rat model for ligature-induced periodontitis. The results demonstrate the formation of healthy periodontal tissue; here, the cells are perpendicular to the cementum and AB, with multiple blood vessels and well-oriented periodontal cells and fibers. Also, the application of AT-MSCs had a lower impact in decreasing inflammatory infiltration in comparison to the control [9] and increased the restoration of the AB [10]. In mice models of bacteria-induced periodontitis, AT-MSCs stimulate cementum regeneration, the organization of PDL fibers and PD vessels, and higher BMP-2 and osteopontin (OPN) expression [78]. In a minipig periodontitis model, AT-MSCs in fibrin gel caused new AB formation, collagen fiber formation and decreased neutrophil infiltration in periodontal defects. Application with matrices such as b-tricalcium phosphate (b-TCP) and human cancellous freeze-dried graft (HCG) were found to stimulate osteogenic gene expression and higher BV and TT [10].

Umbilical cord MSCs (UC-MSCs) are cost-effective and renewable sources of stem cells. The collection of these cells does not involve invasive methods and avoids the ethical debates associated with human embryonic stem cells. Furthermore, UC-MSCs have shown low levels of immune rejection in living organisms and do not form tumors [79].

Many studies have shown that UC-MSCs have a reduced capacity for osteogenic differentiation compared to bone marrow and periodontal ligament MSCs [80]; however, several studies have demonstrated that UC-MSCs can differentiate into odontoblast-like cells in vitro and in vivo [81,82]. This has been proved by findings on the expression of dentin sialoprotein (DSP) and dentin matrix protein-1 (DMP-1) [81]. UC-MSCs are considered in periodontitis therapy mainly due to their high anti-inflammatory potential. UC-MSCs applied with β-TCP bioceramic stimulate the formation of new bone tissues, cementum, and periodontal ligament fibers at 8 weeks after periodontitis [83]. Bone collagen particles with UC-MSCs stimulated bone repair and regeneration in a rabbit model with alveolar clefts [84]. Also, the therapeutic effects of UC-MSCs can be explained by their extracellular vesicles. Exosomes from UC-MSCs can enhance the osteoblastic differentiation of PDLSCs under high-glucose conditions through the PI3K/AKT signaling pathway [85].

The therapeutic potential of each cell source described above has been demonstrated in preclinical animal models for periodontitis treatment, as summarized in Table 1. While the effectiveness of cell-based therapy in animal models is well-established, its translation to human application requires robust clinical validation. Currently, clinical data confirming this efficacy in humans remain limited. For instance, a recent study investigated the direct transplantation of autologous PDLSCs in the treatment of human periodontal defects [86]. It was shown that PDLSCs transplantation resulted in better bone fill compared to the control group. Despite these promising early results, larger clinical trials are necessary before this therapy can be widely applied. Furthermore, critical factors such as donor-to-donor variability must be considered to ensure consistent and predictable clinical outcomes.

## 4. Biomaterial for Periodontal Regeneration (A Brief Overview of Different Types of Biomaterials)

To effectively explore the biomaterials studied for periodontal ligament regeneration, a broader perspective on all biomaterials used in the periodontal area, rather than just those for PDL, is necessary. In this section, we briefly review the biomaterials that can be considered in relation to periodontal regeneration.

Various biomaterials with distinct structural and biological properties have been utilized for periodontal tissue regeneration [90]. These biomaterials may be classified as polymers and additives. Polymers comprise biopolymers and synthetic polymers, each displaying distinct characteristics. Polymers constitute the primary matrix of the engineered tissue intended for transplantation, facilitating either the engraftment or regeneration of PDL. Simultaneously, they ensure effective integration with the AB tissue beneath the PDL and prevent the downgrowth of gingival epithelium to maintain optimal PDL and bone regeneration [91].

### 4.1. Polymers for the Matrix

Biopolymers such as collagen and gelatin can exhibit superior biocompatibility and bioactivity, acting as ECMs by providing specific binding sites and protecting and delivering signaling molecules for periodontal tissue cells; however, they lack mechanical strength and batch variation [92].

Collagen, the main component of the native periodontal ligament, is ideally suited for 3D scaffold preparations that replicate both the soft and rigid tissues of the periodontium due to its fiber-like structure and mechanical stability and flexibility. Research indicates that collagen enhances gingiva health by augmenting gingiva thickness and protecting exposed roots, and it can also serve as a bone-grafting material to facilitate the biomineralization and regeneration of AB compromised or lost due to periodontitis [93].

Gelatin is a collagen derivative that has been used for periodontal regeneration due to its biodegradability via enzymatic degradation without inducing an immune response, biocompatibility that improves cell adhesion, proliferation, and viability, and low toxicity [94]. However, the challenging processing of gelatin hydrogel, caused by its unstable gelation process, limits its applications. Thus, gelatin is conjugated with a methacrylate group to create gelatin methacryloyl (GelMA), which can rapidly crosslink under UV light [95]. Gelatin-based scaffolds are utilized in PDL repair and regeneration, as well as in AB formation, due to their three-dimensional porous architecture, which can be modified through cross-linking conditions to enhance cell adhesion, proliferation, and the delivery of drugs, genes, and growth factors [96].

Chitosan is a biocompatible, biodegradable, and anti-inflammatory natural polymer obtained from chitin, which is extensively utilized in periodontal regeneration. It also exhibits mucosal adhesion and could be metabolized into non-toxic and readily excretable byproducts. Chitosan demonstrates antimicrobial properties, thereby reducing the risk of infection. The structure facilitates conversion into gels, films, and fibers for mechanical support [97].

Hyaluronic acid (HA) is another natural polymer, which is a glycosaminoglycan and exists in the human body, particularly in the skin, AB, and dental cementum [98]. A notable characteristic of HA is its hydrophilicity, and HA-based scaffolds possess high moisture content, which facilitates tissue regeneration. Moreover, due to their viscoelastic properties, HA-based materials can undergo deformation under stress and subsequently return to their original shape upon the removal of that stress [99].

Derived from algae or bacteria, and being widely used for tissue engineering applications, alginate is an anionic polymer that exhibits low toxicity and forms a gel matrix upon exposure to different valence cations [100]. Alginate hydrogel is favored in periodontitis treatment due to its dynamic cross-linking, biocompatibility, and cost-effectiveness; however, it possesses inadequate mechanical properties, excessive softness, significant swelling and shrinking during gelation, and insufficient mammalian cell adhesion; thus, it is often combined with various materials or molecules to augment its functionality [101].

In addition to natural polymers for periodontal regeneration, synthetic polymers are also utilized as the primary component of the structural matrix. Synthetic polymers utilized for periodontal regeneration may demonstrate enhanced mechanical stability and biocompatibility, yet they are deficient in cell-adhesive properties. Synthetic polymers are manufactured industrially from inorganic materials and categorized into absorbable and nonabsorbable polymers. Resorbable polyesters are the most prevalent synthetic polymers, comprising polycaprolactone (PCL), polylactic acid (PLA), polyglycolic acid (PGA), polylactic–polyglycolic acid (PLGA), polyethylene glycol (PEG), and PEG combined with PLGA (PEG-PLGA) [102].

PCL, the most representative FDA-approved synthetic polymer, is a linear bioresorbable aliphatic polyester known for its exceptional thermal stability and moldability. Nonetheless, its hydrophobic nature restricts medium access and drug dissolution and negatively affects cell attachment, proliferation, and differentiation; therefore, surface modifications are essential, although its processability permits utilization in 3D printing for tissue defect repair [102].

PLA and PLGA, which are formed by the copolymerization of PLA and PGA monomers, are additional FDA-approved and biodegradable polymers used in tissue engineering and drug delivery applications [103]. The crystallinity of these polymers affects their mechanical properties, such as swelling, and due to their adjustable mechanical, biological, and degradation characteristics, their application in periodontal tissue engineering has been widely studied [91].

PEG is a hydrophilic, biocompatible synthetic polymer extensively utilized in the biomedical field for drug delivery, tissue engineering, and surface modification [104]. PEG can have adjustable properties and the capacity to create hydrogel scaffolds through photopolymerization. For periodontal regeneration, it can be blended with other polymers to increase hydrophilicity and be used as hydrogels to maintain defect space, deliver growth factors, and provide structural guidance for regenerating periodontal ligament, cementum, and AB [105].

### 4.2. Additives for Periodontal Regeneration Constructs

Additives consist of various materials, including bioactive agents like antioxidants, antibiotics, growth factors, peptides, and nanoparticles. These materials could be added with the purpose of either biological or structural improvement for the final regenerative structure. Additionally, osteogenic additives and agents have been used to enhance bone formation on the part of the engineered structure that comes into contact with the AB to encourage bone integration.

#### 4.2.1. Antibacterial/Antioxidants

Periodontitis, characterized by its high prevalence, is a chronic inflammatory disease induced by bacterial infection. Moreover, excess reactive oxygen species (ROS) generated in chronic periodontitis can directly degrade cellular biological macromolecules through lipid peroxidation, protein denaturation, and DNA damage, thereby disrupting cellular growth and periodic development and causing harm to the host periodontal tissue [106]. Antibiotics and antioxidants used for periodontal regeneration will promote tissue healing by providing antioxidant activity and antibacterial properties, which can result in more efficient tissue regeneration. In addition to using antioxidant polymers such as chitosan for periodontal regeneration, researchers have looked into various natural materials that have antibacterial and antioxidant properties for use in periodontal regeneration. A wide range of antibacterial and antioxidant materials have been used for tissue engineering and regenerative medicine; these materials could have natural sources, such as the ones derived from plants like cardamom, curcumin, and aloe vera or flavonoids such as quercetin, genistein, and silibinin [107]. Examples of synthetic materials with antibacterial and antioxidant properties in periodontal tissue engineering include metals and metal oxides, such as silver and nanoparticles, which could be incorporated into the engineered construct [108].

#### 4.2.2. Growth Factors

Growth factors (GFs) and bioactive agents, especially extracellular vesicles (EVs), are essential in periodontal tissue engineering due to their capacity to regulate cellular recruitment, immunomodulation, and differentiation. Researchers have extensively studied the incorporation of growth factors into periodontal tissue engineering structures recently. For example, basic fibroblast growth factor (bFGF) was the first member of the FGF family to be found and it plays a multifunctional role in cellular and metabolic homeostasis. Due to its potent induction of vascularization, proliferation, and migration, it plays a crucial role in wound healing [109]. Moreover, growth factors such as platelet-derived growth factor (PDGF), fibroblast growth factor (FGF), and vascular endothelial growth factor (VEGF) have been shown to be able to improve angiogenesis, activate endogenous progenitor cells, and improve the synthesis of ECM for periodontal regeneration [110,111].

#### 4.2.3. Nanoparticles

Nanoparticles are nano-dimensional materials that have garnered significant interest among tissue engineers for various applications, including antibacterial and anti-inflammatory immunomodulation, improvement of mechanical properties, and induction of stem cell differentiation [112]. A wide range of nanoparticles have been studied for their potential use in tissue engineering during the last decade; importantly, these can be categorized as metallic- or carbon-based. Additionally, there are ceramic nanoparticles, such as TCP nanoparticles and silica-based nanoparticles.

Metallic and metal oxide nanoparticles can possess different characteristics; for instance, silver NPs and zinc oxide nanoparticles (ZnO-NPs) are antibacterials. Magnesium oxide NPs (nMgO) are also antimicrobial but could induce osteogenesis. AuNPs, in addition to their antimicrobial properties, could induce human PDLSC differentiation [112,113].

Carbon-based nanoparticles, including carbon nanotubes, graphene, and carbon dots, have also been extensively studied for tissue engineering [114]. Carbon-based nanomaterials can either make engineered scaffolds mechanically stronger or induce stem cells’ differentiation into different types of cells, depending on certain conditions [115]. For example, graphene quantum dots (GOQDs) have been shown to effectively promote the osteogenic differentiation of hPDLSCs by regulating mitochondrial dynamics [116]. Carbon nanotubes have also shown antibacterial activity and improved proliferation of hPDLSCs when incorporated into a scaffold for periodontal regeneration [117]. Graphene oxide has been widely studied for bone regeneration, has been demonstrated to induce osteogenic differentiation in stem cells, and has also been used in engineered scaffolds for periodontal regeneration that improve AB regeneration [118].

#### 4.2.4. Inorganic Additives

Besides the mentioned additives, inorganic materials have also been widely used for scaffolds studied for periodontal regeneration. These organic compounds, which can improve the mechanical strength of the structure, are intended to help AB regeneration and its integration with the designed scaffold [119].

Having affinity to bone mineral composition, calcium phosphate (CaP) is bioactive and osteoconductive and is the main inorganic compound used for hard tissue engineering. Hydroxyapatite (HAp), a prevalent CaP graft biomaterial, is recognized for its ability to chemically bond directly to bone, leading to robust osseointegration and exhibiting enhanced regenerative outcomes in clinical metrics such as probing depth and CAL [119]. TCP, especially β-TCP, demonstrates remarkable resorbability, biocompatibility, and osteoconductivity, resulting in probing depth reduction and CAL, although its regenerative capacity is inconsistent [120,121].

Calcium sulfate (CS), exhibiting compressive strength superior to cancellous bone and functioning as a barrier, enhances periodontal regeneration, reduces post-surgical recession, and provides clinical alternatives to membrane placement [119,122].

Bioactive glass, characterized as a CaO, is a SiO_2_-based glass with osteoconductive and osteoinductive properties; these properties are indicated by increased osteogenic marker expression, and produce statistically significant enhancements in PD and CAL. However, human histological evaluations occasionally reveal restricted regenerative results [119,123].

### 4.3. Structural Considerations for Periodontal Regeneration

When working with engineered tissues, in addition to the composition and the materials used for the scaffold or substrate in designing them, it is also crucial to study their structural features. An engineered tissue must not only provide a biological-cell-friendly environment for living cells to thrive, produce ECM, and develop into mature functional tissue, but it must also ensure structural compatibility with the normal behavior and functions of cells to enhance tissue repair efficiently in vivo [124].

#### 4.3.1. Porosity and Permeability

In tissue-engineered constructs, cells also need to communicate with each other and have free spaces to migrate. Moreover, the nutrients need to be homogeneously distributed through channels inside the construct to feed all the cells. Optimal porosity facilitates early microvascular infiltration, graft stabilization, and integration with host tissue; here, macropores (100–700 μm) enhance angiogenesis, while micropores (<100 μm) impede viability due to ischemia [125,126]. Permeability is essential for the transport of oxygen, nutrients, and signaling molecules and must be tailored to address tissue-specific requirements, enabling nutrient diffusion, waste elimination, cell infiltration, and integration while maintaining mechanical stability [125]. The optimal pore size for osteoblast activity in bone scaffolds remains ambiguous, generally ranging from 20 to 1500 μm. Research indicates that smaller pores (~40 μm) facilitate denser cell populations, while larger pores (~100 μm) enhance migration; however, other studies suggest that pores exceeding 300 μm are essential for osteogenesis [125]. PDLSCs were also successfully cultured and proliferated on scaffolds with porosities of 85% and over, with average pore diameters of 162 μm (range, 116–515 μm) and a pore wall [127].

#### 4.3.2. Mechanical Properties

Every specific tissue in the body exhibits distinct mechanical properties. Native cells cultured in vitro on the construct, exhibiting identical mechanical properties, demonstrate enhanced responsiveness, resulting in more typical and mature functionality [128]. The mechanical properties of the engineered tissue are also crucial for in vivo applications, as they must endure the forces encountered. Features such as tensile strength, stiffness, and elasticity are essential mechanical properties that need to be comprehensively assessed to accurately replicate the environment of native tissue. Engineered tissue or scaffolds must be able to withstand mechanical forces during handling and implantation and they must protect the tissue from damage caused by strong mechanical forces, such as tissue tension or occlusion [129]. Energy dissipation in oral structures is essential for attenuating shock waves and reducing stress, requiring enhanced damping properties to improve durability and force resistance. Low modulus materials induce stress concentration, transferring energy to neighboring structures without dissipation during contact [130].

The design of scaffolds for periodontal regeneration must be appropriately aligned to facilitate the transmission of quantitative and mechanobiological signals that connect material properties to cellular behavior and fate. PDL is a unique connective tissue showing anisotropic elasticity and viscoelastic damping, which transmits masticatory forces while preserving homeostasis [24]. Scaffold design for periodontal regeneration should match appropriately to guide quantitative and mechanobiological signals that can link material properties to cellular behavior and fate. PDL is a unique connective tissue showing anisotropic elasticity and viscoelastic damping, which transmits masticatory forces while preserving homeostasis [24]. Optimal scaffolds should thus replicate the native modulus of periodontal ligament (50–150 MPa) while creating gradients towards the more rigid cementum and bone regions (>1 GPa) to facilitate physiological force dissipation [23].

Substrate stiffness is a key factor in mechanotransduction, affecting integrin clustering and cytoskeletal tension that determine PDLSC lineage commitment [131]. Reports indicate that enhanced substrate stiffness significantly impacts mechanotransduction. For example, in one study, the osteogenic differentiation of PDLSCs was enhanced on rigid substrates, with a stiffness range of 6 to 135 kPa. Notch pathway markers were elevated in the PDLSCs cultured on rigid substrates [132]. In another study, where the researchers cultured PDLSCs on a rigid substrate (~56 kPa), they were able to promote osteogenic differentiation; this was evidenced by the elevated RUNX2/OCN levels and the enhanced mineralization, in contrast to those attained using a soft substrate (~15 kPa). These results were reported to be associated with the activation of the ERK1/2 signaling pathway on the rigid matrices, facilitating the nuclear translocation of the mechanosensitive transcriptional coactivator YAP. In a rat periodontitis model, the affected periodontal ligament exhibited significant softening (~23 kPa), which inhibited the ERK/YAP axis and resulted in compromised osteogenesis and alveolar bone resorption. Consequently, the ERK-mediated nuclear translocation of YAP constitutes a crucial mechanotransduction pathway by which matrix stiffness regulates the osteogenesis of PDLSCs [133].

#### 4.3.3. Biodegradation

Another important aspect of engineered tissue/scaffolds is their biodegradation, specifically after being implanted in vivo [134]. In vitro, as cells proliferate and generate ECM, they necessitate space; thus, the scaffold and engineered construct must exhibit biodegradation properties that facilitate the removal of materials to accommodate the expanding cells and their ECM. In vivo, also, the same need exists, as the proliferated cells on the constructs will aim to engraft with host tissue, and host tissue will be integrated with the transplanted structure through sufficient biodegradation [135]. Biodegradation can be influenced by both the composition and the structural/shape design of the scaffolds [134]. For periodontal regeneration, degradable biopolymers, such as collagen, chitosan, and alginate, are used, and synthetic ones, such as PLA, PGA, and PLGA, have biodegradation with hydrolysis, enzymatic, and bulk erosion mechanisms of degradation [136]. For structural design, factors that also influence degradation speed include high surface area, porosity, pore size, and crystallinity; the reasons for this influence are enhanced fluid penetration, accelerated enzymatic/microbial access, and mechanical stress distribution [135,137]. Moreover, the suitable biodegradation speed for periodontal regeneration has been reported to be 6–12 weeks for initial defect filling and 6–12 months for complete tissue maturation [138,139,140].

#### 4.3.4. Structural Alignment

In addition to the biological and mechanical properties of the cellular niche, other factors may also exert influence. The configuration of the native niche presents a significant challenge in replicating the cellular environment [141]. The periodontal ligament, as a connective tissue, is composed of aligned collagen fiber. Thus, PDL cells encounter aligned fibrous structures in their niche [142]. Creating an aligned structure around cells would be a crucial idea to improve their function and mimic native tissue, thereby providing a more biomimetic niche for PDL cells. Thus, a biomimetically aligned fibrous structure enhances the cells’ sense of home and the practical application of the final engineered tissue for in vivo transplantation. It was shown that scaffolds with aligned structure improve PDLSCs differentiation, organized periodontal ligament formation, and AB restoration [142,143]. Alignment of fibers in electrospun or 3D-printed scaffolds has been demonstrated to improve load transfer and facilitate tenogenic or ligamentous differentiation through YAP/TAZ signaling [144].

## 5. Biofabrication Strategies for Periodontal Regeneration

Tissue-engineered scaffolds are actively used for the therapy and reconstruction of periodontal tissue. Modern scaffolds can combine the properties of bone tissue with those of the soft tissue found in the gum and periodontal ligament, due to their multicomponent composition, porosity, and mechanical properties. Biphasic, triphasic, and multiphasic tissue-engineered scaffolds are common constructs to imitate the compartments of alveolar bone, cementum, ligament, and gum. Each compartment of these scaffolds has characteristics similar to native tissue, and the overall structure provides functional structural biomimicry with the correct spatial organization of different cell types after implantation [145]. Thus, modern tissue-engineered constructs for the treatment of periodontal diseases are able to induce osteogenesis while isolating the area of damage and modeling soft tissues and the periodontal ligament. Various biofabrication methods are used to create these complex structures, each with its own pros and cons. Moreover, the production method also affects how exactly cells and other biological components will be introduced into the scaffold.

Here, we provide a brief summary of current achievements in biofabrication for periodontal disease treatment.

### 5.1. Conventional Methods of Biofabrication

The conventional methods for porous scaffold manufacturing include freeze-drying, phase separation, gas foaming, and solvent casting. The common disadvantage of all these techniques is that a practitioner has very limited control over pore size and their distribution within the scaffold [146]. Moreover, these methods mostly use organic solvents to dissolve materials like PCL; in this case, scaffolds need to be purified from residual quantities of these solvents to avoid potential cell death after seeding and inflammatory responses after implantation. The use of organic solvents also restricts seeding biomolecules and cells into the scaffold at the stage of manufacturing; therefore, these methods tend to be more time- and labor-consuming than alternatives [147].

An example of a multi-component scaffold prepared through the freeze-drying method using polyvinyl alcohol, gelatin, and Arabian gum–hydroxyapatite (gum–HA) was provided by Liang et al. The authors tested the antibacterial properties of the final scaffold compositions using different weight percentages of gum–HA (0, 2, 4, 6%). The addition of gum–HA reduced the acidity of the scaffold environment and made it more stable; the composition that included 4% wt gum–HA showed antibacterial properties exceeding the other groups [148]. Huang et al. used triphasic TGF-β3/recombinant collagen/chitosan freeze-dried sponges loaded with hPDLSCs to assess the influence of TGF-β3 on the osteogenic differentiation of these stem cells. Osteogenic differentiation was evaluated by ALP level, and IHC staining indicated that osteoblast marker (Runx2, BMP-2, COL I) expression was higher in cells surrounding defects at 12 weeks after implantation using hPDLSCs-positive triphasic scaffold compared to the other groups [149]. Another study describes the complex manufacturing process of biphasic scaffolds. It combines parallel-aligned concentrated growth factor (CGF) arrays produced by a microstamping technique with porous intra-fibrillary mineralized collagen self-assembly (IMC). CGF/IMC architecture is frozen in liquid nitrogen after assembly and lyophilized. The finalized product exhibited a Young’s modulus of the rigid phase of 1409.00 ± 160.83 MPa, whereas the soft compartment was 42.62 ± 4.58 MPa, successfully utilizing different mechanical properties in one scaffold. This bilayer architecture presented effective recruitment of host stem cells and development of vascularized mature bone island structures in 8 weeks after implantation as well as activation of the TGF-β1/Smad3 signaling pathway [150]. A separate study employed solvent casting with salt leaching to produce PLGA-based scaffolds (wt. 40 mg) incorporating magnesium nanoparticles (wt. 10, 20, 40 mg). The authors found that the PLGA scaffolds loaded with 40 mg of magnesium exhibited higher maximum stress and compressive modulus than the magnesium-free scaffolds (241 ± 84 kPa vs. 79 ± 7 kPa and 2.9 ± 0.7 MPa vs. 0.7 ± 0.1 MPa), while the samples with concentrations of 10 and 20 mg showed lower mechanical strength. Moreover, cell media extracts of the magnesium-loaded PLGA scaffolds (×2, ×4, ×10 dilution) showed enhanced BM-MSCs proliferation compared with the PLGA-only scaffolds. In vivo tests for the PLGA + 10 Mg scaffolds demonstrated no presence of magnesium nanoparticles after 8 and 16 weeks post-implantation, and bone regeneration and mineralization occurred without any signs of inflammation [151].

### 5.2. Decellularized Structures

Decellularized extracellular matrix (dECM) scaffolds are biomaterials derived from human or animal organs and tissues, created by eliminating immunogenic cellular components through decellularization techniques, and they are currently receiving significant attention; dECM has even been shown to have unique immunomodulation effects [152,153]. Decellularized extracellular matrices are employed for periodontal regeneration due to their capacity to maintain the integrity of the natural tissue structure, as well as their inclusion of growth factors and extracellular vesicles [154].

In a study by Farag et al., it was demonstrated that decellularized cell sheets of hPDLSCs contain residual growth factor loads, such as residual bFGF, VEGF, and hepatocyte growth factor (HGF); the amounts of these factors are consistent with the serum concentrations. However, collagen quantification revealed increased collagen content in the decellularized cell sheets that shows no significant effect on the retained collagen, therefore preserving the native structure of the collagen network [155].

Further research highlighted *ALPL*, *BMP-2*, *OCN*, *OPN*, and *VEGF* dynamics after the recellularization of cell sheets with hPDLs. It is stated that this set of factors was undetected at 3 and 7 days after recellularization, while *COL1A1* and *COL1A2* were upregulated at these timepoints, indicating initial rapid proliferation after reseeding. Fourteen days after reseeding, collagen markers were downregulated, while *VEGF*, *BMP-2*, *OCN*, *OPN*, and *TNC* were upregulated, indicating a change in ECM production and the beginning of pro-angiogenic effects. In comparison, human periodontal MSCs in this study demonstrated different *COL1A1*, *COL1A2*, and *COL3A1* mRNA expression profiles; the most significant one was upregulation of *COL1A1* on day 14. It was demonstrated that recellularized cell sheets were superior for PDL tissue engineering than PCL scaffolds, as these constructs promoted periodontal attachment in a rat periodontal disease model [156]. The authors also compared different decellularization protocols for combined cell sheet and PCL membrane constructs. The most effective techniques identified by authors are NH_4_OH/Triton X-100 and DNase treatment, as these methods demonstrated superior efficiency in DNA removal and preservation of structural integrity [157].

Decellularized cell sheets are also common and are usually used in combination with tissue-engineered scaffolds produced by other methods. The decellularized hPDLSCs cell sheets were used with a PCL/gelatin nanofiber carrier structure to obtain the appropriate mechanical strength of the final construct, and 15-deoxyprostaglandin J2 (15d-PGJ2) nanoparticles were added to promote regenerative and anti-inflammatory properties. Collagen I and fibronectin were distributed similarly in hPDLSCs and decellularized hPDLSCs cell sheets. An in vivo experiment with a murine periodontal defect model showed the formation of the new bone both in groups with or without nanofibers 4 weeks following implantation [158].

The advantage of using a decellularized cell matrix is that it can be recellularized and remodeled with new cells supported by the bioactive components that are still present. Ivanov et al. studied the effects of decellularized tissues on osteogenic and odontogenic differentiation in periodontal stem cells. After two weeks of cultivation in decellularized tooth matrix (dTM) and periodontal ligament (dPDL), in all scaffold variants, spindle-shaped cells formed a dense monolayer, and IHC analysis demonstrated the presence of OPN and osteocalcin OCN osteogenic markers. In the 3D culture group using a combination of collagen I with both dTM and dPDL, PDLSCs and periosteum cells had more diverse morphology (elongated, spindle-shaped, and rounded types were observed) than in the monolayer cells and expressed the odontogenic marker dentin sialophosphoprotein (DSPP). Thus, the microenvironment of dTM and dPDL promoted both pathways of differentiation [159]. Furthermore, the authors evaluated the osteogenic and odontogenic differentiation potential of individual components of the ECM (HA, fibronectin, laminin). The osteogenic (OCN, OPN, ALPL) and odontogenic DSPP markers were used to measure the differentiation potentials in PDLSCs cultured in collagen I hydrogel with dECM, a combination of dTM and dPDL. Only the groups with dECM expressed DSPP, while the “stemness” factors CD44 and STRO-1 were present in all groups except those that were Lam-positive. Osteogenic factors (OCN, OPN) were upregulated in dECM-positive groups. The composition, including collagen I, dECM, and fibronectin, showed the superior differentiation potential of PDLSCs toward osteoblasts and odontoblasts compared to other groups [160].

Another study compared two different decellularization protocols: Protocol I, which used NH_4_OH (0.1%) and 2% Triton X-100 for 72 h, and Protocol II, which involved 1% SDS for 24 h followed by 1% Triton X-100, in relation to the subsequent repopulation of hPDLSCs. The integrity of decellularized tissue was evaluated by the density of Sharpey’s fibers. Both protocols lead to fibronectin elimination in ECM; moreover, the osteogenic markers (ALPL, COLXII, CP23, OCN) were significantly upregulated after cell reseeding. The main difference between protocols lies in fibronectin and Col XII expression levels; Col XII was enhanced for protocol II, while fibronectin expression was reduced. It is stated that Col I expression decreases with tissue maturation, while Col XII expression is enhanced in mature tissue, and it plays a role in the alignment of collagen fibers [154,161].

However, despite the advantages of dECM scaffolds, the decellularization approach has some limitations. Complete decellularization is unachievable and residual DNA or RNA content can be immunogenic, so different techniques are tested to reduce the possible immune response [162]. And the issues of standardization and scale-up of decellularization protocols are still present mostly due to the variety of protocols and donor tissues and lack of commercially available specialized decellularization devices. Decellularized matrices (dECMs) for periodontal regeneration hold considerable potential but require thorough examination through explicit risk assessment and quality control measures. Even the most effective decellularization techniques cannot completely eliminate all cellular components; remaining DNA, RNA, or membrane fragments may elicit immune responses [163]. Generally, numerous studies regard < 50 ng DNA per mg dry weight and fragment lengths < 200 bp as acceptable thresholds, with the absence of visible nuclei under DAPI/H&E serving as further validation [164]. Residual detergents, such as SDS and Triton X-100, can alter collagen architecture by either stiffening or weakening the structure. This process changes the mechanical signals that PDLSCs receive, which could cause them to differentiate incorrectly. To confirm that extracellular matrix components (like collagen and glycosaminoglycans) are still present, it is necessary to follow strict washing procedures and to perform proteomic analyses. Inconsistencies become even more critical when donors are different, tissue is thicker, and protocols are different. Researchers have looked into automated and perfusion-based decellularization systems to reduce batch variation and improve reproducibility [165]. To guarantee the clinical relevance of periodontal dECM, every batch must undergo standardized quality control evaluations, including the quantification of residual nucleic acids, the preservation of ECM protein profiles, the assessment of mechanical integrity, and screening for immunogenicity, such as macrophage cytokine release [166].

### 5.3. Bioprinting and 3D Printing

Non-biological 3D printing employs many strategies that are able to create complex structures for application in tissue engineering but have some limitations for including cells directly into the printing process. The most popular method of 3D printing is fused deposition modeling (FDM), which uses continuous extruded filament of melted polymer material to form the structure layer-by-layer, fusing adjacent layers during the process of printing. However, the high temperature of the filament is a major factor that restricts the use of cells during the printing process [167]. Despite this, FDM has applications in tissue engineering, using biocompatible materials like PLA or PCL to fabricate scaffolds for future cell seeding [168,169].

Another 3D printing strategy is selective photopolymerization, which includes methods such as stereolithography (SLA)—a technique for selective layer-by-layer photocuring of liquid resin with a laser beam. Direct-light processing (DLP) is another method of selective polymerization using a projector instead of a laser, resulting in faster printing speed [170]. Two-photon polymerization—a state-of-the-art approach using a near-infrared femtosecond laser to induce two-photon absorption, resulting in microscale polymerization—is used to produce microstructures for a wide range of applications, such as drug delivery, microfluidics, and tissue engineering [171].

Three-dimensional bioprinting is a modern computer-assisted method of biofabrication, which involves the layer-by-layer formation of mechanically stable structures from various biomaterials, with the possibility of introducing cells and other biologically active components inside at the printing stage [172]. To date, various methods of three-dimensional bioprinting have been developed, which differ in the printing method itself, the accuracy of structure formation, and the requirements for materials [172]. For example, extrusion bioprinting involves layer-by-layer application by extruding bioink. This method is compatible with a wide range of materials but requires the optimization of printing protocols due to difficulties in achieving high printing resolution for each specific bioink composition. It also struggles to achieve high-resolution structures and is limited to 200–1000 μm [173]. DBB (droplet-based bioprinting) is another type of bioprinting. This is a high-resolution additive manufacturing technique that creates intricate biological structures using carefully regulated, picoliter-volume bioink droplets. DBB is characterized by its high-throughput, non-contact deposition ability, allowing exceptional control over cell positioning and the patterning of multiple biologics with minimal mechanical stress [174]. Inkjet (thermal, piezoelectric, and electrostatic), acoustic, acoustophoretic, micro-valve, and electrohydrodynamic bioprinting are among the sub-modalities of the technology. Each of these uses distinct physical mechanisms, such as electric fields, pressure pulses, and acoustic waves, to create and release droplets [174]. The bioprinting approach for treating periodontal disease is relatively new, but it has already been demonstrated to have some promising results [94]. Bioprinting requires meticulous optimization due to the complexities of hydrogel bioink behavior and interaction with cells and other tissue building blocks (e.g., organoids, cell spheroids, etc.). It was shown that bioprinted hydrogel composed of gelatin methacryloyl (GelMa), sodium alginate (SA), and bioactive glass microspheres (BGMs) influenced osteogenic-related genes OPN, OCN, ALPL, Runx2, and COL I at days 4, 7, and 14 after mBMSCs seeding. Shortly, the GelMa/SA/BGM group exhibited a higher expression of the mentioned osteogenic genes compared to the GelMa/SA group, while the GelMa/SA group induced a higher expression than that of the control group without scaffolds [175].

Liu P. et al. designed a mix of SA and gelatin containing acellular dermal matrix (ADM) for oral soft tissue regeneration. Two groups of ADM/gelatin/SA scaffolds were made, laden with spindle fibroblast morphology and oval fibroblasts (control). In the 14-day period, COL1A1 expression was higher in the spindle group, while PECAM1 and VEGF-A were slightly higher than in the control group [176]. In the next step, the ADM scaffold loaded with gingival fibroblasts demonstrated better regenerative capabilities after implantation on an in vivo model of a canine periodontal defect in comparison with the ADM-cell-free group. Keratinized gingiva increment in the ADM cell-containing group was 0.58–1.93 mm, and the attached gingiva increment was 1.83–2.30 mm against 0.67–1.30 and 0.83–1.30 mm, respectively, in ADM cell-free scaffolds [177].

Fiber alignment is one of the key considerations in biofabricating bone–ligament scaffolds, and bioprinting is used to address this issue [178]. For example, GelMa and dECM bioink were used to produce a bioprinted scaffold, encapsulating cells and providing favorable topographical and biochemical environments. Their effectiveness was demonstrated by cell viability tests, with high amounts of early osteogenesis markers, ALP and Runx2, as well as a late marker, OCN, for dental follicle cells being encapsulated in the gels. In this study, two types of additive manufacturing were deployed at once; an extrusion-based bioprinter was used to fabricate a grid-like structure for alveolar bone regeneration, while DLP printed a lattice-type module for periodontal ligament regeneration. The scaffold reduced local inflammation by suppressing the release of pro-inflammatory factors by M1 macrophages in the Sprague Dawley rat model [179].

Bioprinting also coexists with techniques of 3D printing for periodontal regeneration. One example is a study by Pilipchuk S. et al., who 3D printed complex molds to produce micropatterned PCL film with grooved pillars (groove width groups: 10, 25, and 50 um), modeling the “ligament” region in combination with the dentin layer. The second part of this integrated scaffold was a model “bone” made of PCL containing 5% HAp and using selective laser sintering (SLS) printing technology. The “ligament” part was seeded with human PDLSCs, while the “bone” contained gingival fibroblasts transduced with AdBMP-7 genes. At week 6, after subcutaneous implantation into a murine model, the samples with 30 μm groove depth showed a significantly affected collagen fiber orientation and improved cell alignment, which indicates that the scaffolds successfully mimicked the native tissue’s architecture [180].

Another application of 3D printing methods for PDL treatment is given by Kim M.G. and Park C.H., who used computer-aided design (CAD) software (Solidworks 2020, Dassault Systems SOLIDWORKS Corp., Waltham, MA, USA) to design the architecture of microgroove patterns and then used 3D wax printing to fabricate specialized molds. The molds were divided into four groups with different slice intervals, including 25.40 µm, 19.05 µm, 12.70 µm, and 6.35 µm. PCL scaffolds were made using these molds. After 7 days of hPDLSCs cultivation on the scaffolds, the cell alignment cultures on the 25.4 µm samples were higher than those of the other groups, and the 6.35 µm sample showed random cell orientation [181].

In 3D printing and bioprinting approaches for periodontal scaffolds, the architecture of filament layout, pore geometry, and strut orientation directly dictates internal load paths and hence the mechanical cues delivered to resident cells. For example, lattice, wavy, or hexagonal infill patterns in bioprinted scaffolds lead to markedly different stress distributions under compression or tension—some struts act as load bearers while others act as shear bridges—and this has been demonstrated in predictive finite-element models of bioprinted constructs [182]. Meanwhile, experimental work confirms that mechanical properties (modulus, stiffness anisotropy) of 3D printed scaffolds depend heavily on macroporous mesostructures (filament spacing, orientation) rather than solely on bulk material chemistry [183]. In the periodontal milieu, directing force through well-aligned struts that mimic natural PDL fiber orientation can help align local collagen deposition and guide PDLSC mechanotransduction. Indeed, multi-compartment fiber-guiding scaffold designs in periodontal defect models have shown improved PDL fiber orientation and more perpendicular insertion to the root surface, consistent with more physiological load paths [184]. When cells adhere to scaffold struts, integrin-mediated focal adhesions sense local strain gradients and transmit forces to the cytoskeleton, which in turn modulate downstream ROCK/YAP/TAZ activation and ultimately direct differentiation toward ligamentous or osteogenic lineages [185,186]. The scaffold’s stiffness and topology modulate local strain magnitudes and spatial heterogeneity in these cues; in 3D and viscoelastic environments, the time-dependent stress relaxation behavior further influences how cytoskeletal tension, nuclear deformation, and gene expression unfold [187,188]. Models of YAP/TAZ spatial mechanotransduction confirm that adhesion patterns and stiffness gradients result in non-uniform nuclear translocation of these factors [189,190].

### 5.4. Electrospinning

Electrospinning is a highly regarded and promising technique in tissue engineering, owing to its capacity to generate fibers ranging from micro–nanometer scale with an extensive surface area [191]. In this biofabrication method, a charged, solubilized polymer is ejected from a syringe needle toward an inversely charged electrode used as a collector. The extrusion itself is caused by electrostatic force overcoming surface tension and forming thin jets of material, producing a non-woven nanofiber mesh on the collector. Thus, the nanofibrous structure of the native extracellular matrix is reproducible with the electrospinning technique. The porosity level and the diameters of nanofibers can be fine-tuned by adjusting the polymer concentration and voltage; therefore, scaffolds of various characterizations can be designed according to the specific needs of the application [191]. For example, Vaquette et al. demonstrated the impact of collector patterning on the morphology and pore size of electrospun scaffolds; it was shown that an increase in pore size from around 10 µm for random-patterned scaffolds to 50 µm for large, round-patterned scaffolds was crucial for cellular colonization by NIH 3T3 fibroblasts within the scaffold [192]. Variations in the speed of the drum collector also affect the alignment of electrospun fibers; it was shown in a comparison of a 100 rpm randomly oriented PLGA layer and a 2000 rpm-aligned PLGA layer that aligned scaffolds have a smaller diameter of fibers and higher mechanical stability [193].

Biphasic scaffolds can contain electrospun elements as carrier structures for PDL cell sheets. In these cases, cell sheets made of PDLSCs are used to substitute the damaged periodontal tissue due to ECM composition, while electrospun scaffolds support the structure and provide space for cell colonization and vascularization [194]. In some studies, CaP is used to increase bone mineralization in biphasic scaffolds and improve their regenerative potential. Dan et al. compare three groups of CaP-PCL electrospun scaffolds seeded with PDLSCs, alveolar bone cells (ABCs), and gingival-margin-derived cells (GMCs) and describe the regenerative potential of these scaffolds four weeks after implantation in a rat periodontal defect model. PDLSCs and ABC-seeded scaffolds in this study successfully formed PDL-like structures as well as new bone and cement, which was demonstrated with sialoprotein and OPN immunohistochemical staining [66]. It was shown that the incorporation of CaP increased osteoinductivity in FDM electrospun scaffolds combined with cell sheets, which was confirmed by SEM visualization and ALP activity tests [195]. Cementum formation on the similar biphasic scaffold without a CaP layer was revealed by the CEMP1 immunochemistry staining, and dentin coverage with cell sheets was around 65 ± 22% compared to the group without cell sheets, 33 ± 4% [196]. In another study, electrospun PCL–chitosan scaffolds with and without ECM were compared to assess their ability to enhance osteogenic differentiation in seeded PDLSCs for the treatment of alveolar bone. It was confirmed that PDLSCs cultured on ECM-containing scaffolds had increased calcification and ALP activity [197].

Electrospun scaffolds have tunable fiber alignment, which can greatly influence cell adhesion and migration [198]. Staples et al. used the 100 μm spaced channels and a pore size gradient of the biphasic scaffold to replicate native tissue alignment perpendicular to the dentin layer. Deep integration between the dentin surface and the bone compartment in the scaffold via guided collagen fibers was confirmed by assessment of nuclei, actin, and collagen I alignment [199]. It was demonstrated that grid-patterned and aligned electrospun scaffolds influence the surface-regulated behavior of PDLSCs by activating yes-associated protein (YAP)-mediated mechanosensing and, consequently, enhancing PDL reform after implantation. Moreover, the topography of the scaffold also affected CD105, periostin, osteopontin, and vinculin [144]. In another study, the triple-layered structure (TLS) was fabricated of gelatin/PCL using a polyporous structure in between two aligned layers to replicate ECM. TLS was later functionalized by BMSC and PDLSC-specific ECM and showed better bone regeneration, vascularization, and ligament fiber alignment than TLS modified with single specific ECM [200].

Besides the advantageous features of electrospun structures, organic solvents used in electrospinning are often cytotoxic, and produced meshes need an additional step of washing [201]. Moreover, material composition for electrospinning is limited, and not all the polymers have an applicable spinnability in popular solvents. Another challenge is to achieve topographic structures and mechanical properties close to those of native tissues, so the studies describe different techniques for adjustable alignment and pore size more beneficial for cell repopulation of scaffolds [192]. Addressing the mechanical properties of these materials, the main issue with electrospun scaffolds is the low load-bearing capacity produced by conventional electrospinning [202].

To conclude, a wide variety of PDL scaffold manufacturing methods are available; however, the conventional methods tend to be less controllable and accurate compared with the latest applications of electrospinning and 3D printing approaches. The progress in the biofabrication field involves using CAD and computer-aided manufacturing (CAM) for personalized treatment and the combination of two or more biofabrication techniques and materials within one scaffold, so these complex bi-/multiphasic hybrid structures have more predictable porosity and architecture, better resorption, and superior mechanical properties compared with the preceding monophasic scaffolds [203]. Thus, future scaffolds for personalized PDL treatments tend to mimic hard and soft tissues simultaneously, with higher precision than conventional techniques, and include cells or cell sheets to enhance regeneration of the damaged tissues.

## 6. Mechanical Stimuli in Periodontal Ligaments Formation

Mechanical stress is crucial to the normal function of periodontal ligaments. PDLSCs respond to mechanical stress by producing a large number of inflammatory cytokines and chemokines, which play an important role in AB remodeling [15]. Moreover, PDLSCs are crucial for preserving the homeostasis and integrity of dental tissue; their involution and atrophy are linked to the lack of consistent mechanical stimuli. Conversely, exposure to excessive forces causes an imbalance between osteogenesis and osteoclastogenesis [204]. Several studies have examined the response of PDL to mechanical forces. For example, it has been demonstrated that, under mechanical stress, collagen bundles are thick, densely organized structures with a relatively large cross-section (~19.2 ± 3.5 microns) [205]. On the other hand, in the absence of mechanical stress, collagen fibers became disorganized and thin, with a smaller cross-section (~5 ± 1.8 microns), and a large number of osteoclast-like cells appeared on the surface of the AB in the area of the horizontal fibers. In both cases, PDL fibroblasts retain their spindle-shaped morphology and remain oriented parallel to collagen bundles; however, under mechanical stress, they develop numerous small processes (e.g., filopodia) which are atypical under physiological conditions [205]. The activator of the kappa-Β nuclear factor ligand receptor (RANKL, or TNFSF11), the kappa-B nuclear factor activator receptor (RANK), and TNFRSF11B (OPG) play a crucial role in regulating these processes [204].

It is also known that PDL provides mechanical stability and acts as a shock absorber, protecting the tooth and AB from potential damage during the chewing process [30].

As is well known, mechanical forces play an important role in regulating the fate and behavior of stem cells [15]. For example, it has been described that so-called “long-term” quiescent hematopoietic stem cells (HSCs), responsible for self-renewal and maintaining the population, are located in the endosteal niche, which is mechanically a stiff niche with a Young’s modulus of 40–50 kPa. At the same time, “short-term” HSCs, actively undergoing differentiation, are characteristic of the perivascular niche, which is rich in endothelial and stromal cells. The Young’s modulus of this niche is significantly lower—about 3 kPa [206]. Meanwhile, the participation of various mechanical stimuli in the “fate” of PDLSCs, as well as their roles in differentiation and bone tissue remodeling, remains poorly studied [15]. The main types of mechanical influences experienced by periodontal ligament cells are stretching, compression, and shear stress.

The key primary sensors in PDLSCs are the mechanosensitive ion channel Piezo1 and the transcription co-activators YAP/TAZ. Studies indicate that mechanical stretching increases Piezo1 expression, leading to an influx of calcium ions (Ca^2+^), activation of CaMKII, and subsequent osteogenic differentiation through the upregulation of Runx2 and ALP [207]. Additionally, tension directly induces nuclear translocation of YAP, which is necessary for the expression of mineralization and periodontal tissue integrity genes. These data support the existence of the Piezo1/Ca^2+^/YAP pathway in PDLSCs, serving as a direct “sensor-to-core” mechanotransduction route [208,209].

The decellularization process can significantly alter the ultrastructure and biomechanical properties of the native ECM, which directly affects the subsequent behavior of reseeded PDLSCs [210,211]. For example, decellularization can affect the stiffness and architecture of the matrix. Aggressive methods (such as those using ionic detergents) may decrease stiffness and disrupt collagen fiber alignment, as well as destroy glycosaminoglycans [212]. PDLSCs are mechanosensitive cells; they respond to changes in the mechanical state of their environment, which can influence their differentiation. Research indicates that PDLSCs cultured on more rigid substrates demonstrate improved osteogenic differentiation. Increased substrate stiffness promotes the activation of ERK1/2 pathways and nuclear translocation of YAP. Inhibition of ERK1/2 reduces YAP nuclear localization and suppresses the expression of osteogenic markers. PDLSCs perceive mechanical signals through integrin receptors, which transmit mechanical stress to the cytoskeleton. On softer, decellularized matrices, cells cannot generate sufficient cytoskeletal tension, leading to sequestration and inactivation of key mechanosensors, i.e, YAP/TAZ. As a result, the YAP-TEAD complex cannot activate pro-osteogenic transcriptional programs, diverting cellular differentiation away from the osteogenic pathway [133]. Moreover, aggressive methods primarily damage fibronectin, but they still also harm collagen XII, although its network may remain more visible against the complete absence of fibronectin [212]. The loss of fibronectin deprives cells of their “scaffold.” The orientation of fibronectin fibrils is established at the earliest stages of cell adhesion and determines the direction for the entire future architecture of the matrix [213]. While gentler protocols better preserve bioactive fibronectin, they may be less effective at maintaining mature, aligned collagen XII—fibril-associated collagen with interrupted triple helices (FACIT)—which regulates the organization and mechanical properties of larger collagen I fibrils. Degradation of Col XII disrupts the micromechanical environment, further impairing the cells’ ability to properly sense and respond to topographical signals [212,214]. As a result, decellularization creates a deficit in mechanical instructiveness: the resulting scaffold is unable to provide the precise stiffness and topographical cues necessary to direct PDLSCs’ fate through canonical mechanosensitive pathways such as YAP/TAZ signaling and integrins.

**Figure 2:**
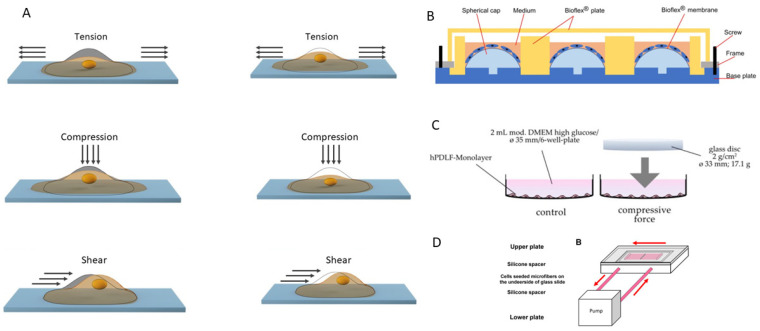
Examples of bioreactors used for mechanical stimulation of cells. (**A**) Types of mechanical forces applied to cells. (**B**) BioFlex bioreactor for tension of cells, growing on membrane. The images were adapted and changed from [16] according to http://creativecommons.org/licenses/by/4.0/ (accessed on 29 August 2025). (**C**) Compression bioreactor to apply compressive force on cultured cells with a sterile glass cylinder. The images were adapted and changed from [215] according to http://creativecommons.org/licenses/by/4.0/ (accessed on 29 August 2025). (**D**) Shear stress bioreactor representing set-up of the parallel-plate flow chamber. The images were adapted and changed from [216] according to http://creativecommons.org/licenses/by/4.0/ (accessed on 29 August 2025).

Bioreactors are used to exert specific effects on these cells, with some models illustrated in Figure 2.

It has been shown that mechanical forces induce dynamic changes in the proliferative potential of PDLSCs, which subsequently leads to tissue remodeling [24]. In the context of mechanotransduction, biological properties, mechanical properties, and biomechanical signal transduction are three main components of this process [15].

Sources in the literature contain information on the analysis of the effects of mechanical stimuli on PDLSCs, specifically regarding gene and protein expression, as well as the secretion of various cytokines and signaling molecules by the cells [16,217]. Mechanical signals have been demonstrated to significantly impact the development of inflammatory processes in PDLSCs. For example, there are studies describing the role of cyclic stretching in the development of complex inflammatory processes. In particular, Sun C. and colleagues demonstrated that an increase in the production of pro-inflammatory cytokines such as interleukin 1β (IL1β), tumor necrosis factor alpha (TNFα), interleukin 6 (IL6), interleukin 8 (IL8), and prostaglandin E2 (PGE2) confirms the presence of an inflammatory response triggered by mechanical impact on PDLSCs [16]. The literature also reports that IL6, PGE2, prostaglandin–endoperoxide synthase 2 (PTGS2), TNFα, IL8, and IL1β play roles not only in inflammation development but also in bone tissue resorption [18,218]. Additionally, IL6 is known to be a key participant in osteoclastogenesis, likely related to the RANKL signaling pathway [18,218,219]. Studies have shown that IL6 levels increase within 24 h after the onset of stretching, with the degree of cytokine secretion correlating with the intensity of mechanical stimulation. However, after 72 h of stretching, increased IL6 secretion is no longer observed [16]. Conversely, other research indicates that changes in the duration of cyclic stretching do not significantly affect IL6 production; instead, the intensity of stretching influences cytokine secretion. For example, gene expression of *IL6* increases with a stretch intensity of 10% over 12 h but decreases twofold at lower intensities of 1% and 5% over the same period compared to controls without stretching [19]. It is also known that mechanical compression leads to increased expression of pro-inflammatory cytokines such as *IL6*, *IL8*, and *cyclooxygenase-2* (*COX2*) after 24 h. This type of mechanical impact also elevates VEGFA, likely related to angiogenic processes [219].

Moreover, the type of compression—static or dynamic—also influences cytokine secretion. Gauthier R. and colleagues demonstrated that static compression of PDLSCs attached to a fibrous PCL matrix resulted in increased IL-6 secretion from day 10 to day 21 of observation. Conversely, dynamic compression resulted in a reduction in IL-6 secretion during the same timeframe. On day 21 of the experiment, IL-6 concentrations were approximately similar under both types of compressive stimuli [220].

Some researchers have suggested that low-force mechanical stimuli induce anti-inflammatory cytokine production, whereas high-intensity stimuli promote pro-inflammatory cytokine secretion. For instance, Wada S. and colleagues demonstrated that stretching cells by 15% for 24 h increases IL6 production [18]. Moreover, *COX2*—a key gene activator of inflammation—showed a 31.5-fold increase in expression under static stretching at an intensity of 10%, compared to controls; no such increase was observed at lower stretch levels of 1% and 5% [19]. In Sun C.’s study, maximum *IL6* expression was observed within the first day at a stretch intensity of 15%. Over time or at lower stretch forces, IL6 levels returned to baseline levels similar to controls. A similar pattern was noted for *PGE2* gene expression: the highest levels were detected on day one during a stretch at 15% [16]. Additionally, there is evidence implicating the toll-like receptor 4 (TLR4) signaling pathway in inflammatory responses induced by mechanical stimuli on PDLSCs. Specifically, it has been shown that TLR4 is activated via high-mobility group box (HMGB1) released during compression, which subsequently triggers NF-κB signaling, which is mediated by myeloid differentiation primary response gene 88 (MyD88); this leads to innate immune responses through the production of various mediators, including inflammatory cytokines [219]. All these findings indicate that there should be further research into patterns governing inflammatory cellular responses to different types and intensities of mechanical stimuli on PDLSCs [16].

As was mentioned earlier, osteogenic differentiation of PDLSCs plays a significant role in the remodeling process of the periodontal complex. It is known that various molecules mediating osteoclast and osteoblast formation are activated at different stages of bone remodeling, and mechanical stimuli contribute to changes in the expression of several genes responsible for these processes [16]. Some of the key genes involved in regulating osteogenic differentiation include transcription factors *RUNX2*, *Osterix* (*SP7*), *ALPL*, *OCN*, *OPN*, *RANKL, OPG*, and several others [16,18]. RUNX2 is one of the key regulators of bone remodeling, activated both in young pre-osteoblasts and in immature osteoblasts; however, its expression level significantly decreases in mature osteoblasts [18]. Research has shown that the expression of *RUNX2* and *ALPL* markedly elevates within 24 h following the initiation of stretching PDLSCs. However, it has also been shown that, by days 2–3, the expression of these genes decreases and ultimately returns to control levels. Membrane stretching at an intensity of 3% results in the highest gene expression levels. Mechanical stimulation at higher intensities does not produce such a significant effect [16]. According to results from another research group, increased *RUNX2* expression was observed at both 12 h (1.52 times) and 24 h (1.98 times), as well as at 48 h (1.38 times) after the start of stretching. Thus, the expression of this gene differed from the control group already after 12 h, increased after 24 h, and decreased after 48 h [17]. Other data indicate that increased *RUNX2* expression was detected three days after initiating cell stretching cultured in pro-osteogenic differentiation medium [217]. These events are likely related to RUNX2’s ability to participate in osteoblast differentiation via the phosphatidylinositol 3-kinase-Protein Kinase B (PI3K/AKT) signaling pathway.

Moreover, the literature reports an increase in phosphorylated Protein kinase B (pAKT) levels under compressive forces on PDLSCs, indicating activation of the AKT-associated signaling pathway under such mechanical stimuli [219,221]. Some studies also suggest that mRNA expression levels of *epidermal growth factor receptor* (*EGFR*) and *fibroblast growth factor 5* (*FGF5*) influence this signaling cascade [221]. It is also known from literature that *ALPL*—the gene encoding alkaline phosphatase—exerts an inhibitory effect on OPG [222]. Changes in OPG levels during cyclic stretching of periodontal ligament cells promote the stimulation of OPG-mediated bone formation [222,223]. Furthermore, under hypoxic conditions, PDLSCs show increased *ALPL* expression and decreased OPG level—information that could be relevant for therapies targeting periodontal tissue disorders since orthodontic tension can induce hypoxic conditions in PDLSCs and thereby stimulate bone remodeling [223].

It was also shown that ALP activity can vary based on the type of mechanical compression force applied. For instance, in the case of dynamic compression, PDLSCs cultured on a fibrous PCL matrix exhibit a decrease in ALP activity, whereas static compression results in an increase. However, on day 21 of the experiment, ALP activity under both static and dynamic compression conditions was approximately similar [220].

A high level of expression of hypoxia-inducible factor 1-alpha (HIF-1α), characteristic of hypoxic conditions, significantly influences bone remodeling processes. Specifically, it reduces the differentiation potential of osteoblasts toward osteoclasts and suppresses their activity. The gene *OPG*, involved in regulating osteoclast activity, interacts with RANKL and receptor activator of nuclear factor-κ B (RANK) to modulate osteoclastogenesis [224]. Under mechanical compression, an initial decrease in *OPG* expression is observed after 3 h of exposure, followed by a subsequent increase [60]. OPG acts as an antagonist to RANKL, preventing its binding to RANK and thereby inhibiting osteoclast formation, shifting the balance toward bone remodeling [225]. The interactions in the RANK–RANKL complex activate signaling pathways such as NF-κB, the mitogen-activated protein kinase family (MAPK), tyrosine kinases c-Src, and PI3K via TNF receptor-associated Factor 6 (TRAF6) [226]. Additionally, Ca^2+^/calmodulin-dependent kinase II (CaMKII) plays a role not only in overall bone tissue remodeling but also in regulating osteoclastogenesis through RANKL and cAMP-response element binding protein (CREB) [60]. Gene *Osterix* is essential for the differentiation of pre-osteoblasts into mature osteoblasts. According to the literature, Osterix expression is inversely correlated to the duration and intensity of mechanical stimulation; here, low-intensity stretching promotes a more significant increase in its expression [16].

RUNX2 stimulates the proliferation of immature osteoblasts, while Osterix inhibits this process. RUNX2 induces Osterix expression, allowing cells to transition from RUNX2+ to RUNX2+ Osterix+ pre-osteoblasts. Subsequently, through the canonical Wnt signaling pathway, these cells differentiate into immature osteoblasts expressing collagen type I alpha 1 chain (Col1a1) and OPN (RUNX2+ Osterix+ OPN+) [227]. Changes in the expression level of the *Col1a1* gene are characteristic of PDLSCs under mechanical stress: stretching increases its expression by 4.4 times compared to control cells, while compression decreases it by 3.7 times [59]. The *OCN* (*BGLAP*) gene also shows altered expression under mechanical stimuli: increased expression is observed as early as 24 h after exposure, reaching a peak at 72 h; maximum expression occurs at a stretch of 10% [16,228]. It has also been demonstrated that the peak transcription level of the *OCN* gene gradually increases by the third day of mechanical stimulation, with the highest expression observed at 10% stretch [16]. Additionally, stretching decreases the expression of genes such as *NOG* and *SFRP4*, creating favorable conditions for osteogenic differentiation. This is because NOG exerts an inhibitory effect on osteogenic differentiation in PDLSCs. Similarly, the *SFRP4* gene not only influences this type of differentiation but also inhibits proliferative activity of periodontal complex endothelial cells [229]. There are also studies focusing specifically on the expression of the aforementioned *OPN* gene—a marker of osteogenic differentiation. For example, it was shown that *OPN* expression increased at 12, 24, and 48 h by factors of 1.47, 1.40, and 0.88 times, respectively [17].

In addition, Phothichailert S. and colleagues demonstrated that PDLSCs cultured on the decellularized extracellular matrix (dECM) of PDLSCs, obtained under intermittent compressive force (ICF), show decreased expression of the *OPN* gene. Similarly, the expression of the *Dentin Matrix Protein 1* (*DMP1*) gene, which encodes a protein important for mineralization and is involved in osteoblast differentiation, is also reduced [230,231].

Moreover, this type of mechanical stimulation led to an increase in the expression level of *Bone Morphogenetic Protein 2* (*BMP-2*) [231]. As is well known, BMP-2 is a participant in the Smad signaling pathway, which triggers SMAD proteins to translocate to the nucleus and activate genes involved in bone formation [232].

Along with genes associated with osteogenic differentiation, it is critical to recognize that changes in gene expression caused by mechanical stimulation affect not only genes involved in cell differentiation but also genes responsible for inflammatory responses, including *FOS*, which is associated with mechanosensation. According to research, the expression of this gene decreases during the first day and increases by the third day of PDLSC’s stretching, with no significant differences observed in transcript levels at different stretch intensities [16].

Mechanical stimulation can induce alterations in cellular components and other cellular behavior. Cytoskeletal changes in cells represent another notable effect of mechanical stimuli. It has been described that both cyclic stretching and compression of PDLSCs lead to an increase in the expression of filamentous actin (F-actin) [20,215]. These cytoskeletal modifications during cyclic stretching are likely related to changes in the expression of phosphorylated Myosin Light Chain 2 (p-MLC2), which is important for actomyosin cytoskeleton dynamics. It has been demonstrated that this type of mechanical stimulus temporarily reduces actomyosin contractility, probably due to a decrease in the actin–myosin cross-bridges necessary for contraction, leading to the reorientation and morphological adaptation of cells [20,233]. Additionally, the literature reports indicate morphological changes in cells subjected to such mechanical stimuli, i.e., cultures not exposed to stretching maintain their typical shape; in contrast, stretched cultures undergo significant reorganization—cells become elongated and oriented perpendicularly to the direction of stretch. This phenomenon suggests the occurrence of active cytoskeletal remodeling, demonstrating that periodontal tissue cells can respond to mechanical load through self-reorganization. Such adaptive reactions play an important role in maintaining mechanical stability and functional integrity of the periodontal complex [20]. As mentioned before, mechanical stretching can stimulate cytoskeletal remodeling in cells, and this process is likely mediated by the Rho/ROCK/non-muscle myosin II (NMMII) signaling pathway, which promotes nuclear translocation of YAP. Studies have shown that treatment with Y-27632 (20 μM)—a specific ROCK inhibitor—and blebbistatin (20 μM)—an inhibitor of NMMII ATPase activity—significantly reduce the cyclic nuclear localization of YAP after stretching. This finding suggests that cytoskeletal changes play an important role in activating YAP in PDLSCs previously subjected to stretching. Additionally, culturing cells under cyclic stretch in an osteogenic medium in the presence of these inhibitors results in decreased ALP activity, further confirming the importance of cytoskeletal remodeling for osteogenic differentiation. Moreover, the expression levels of osteogenesis-related genes such as *OPN*, *RUNX2*, *COL1*, and *ALPL* are reduced after treatment with these inhibitors. These findings confirm that cytoskeletal remodeling is a key process involved in osteogenic differentiation associated with cyclic stretch, with YAP activity being a crucial participant in this mechanism [217]. Furthermore, increased expression of long non-coding RNAs (lncRNAs) *CYTOR*, *MIR22HG*, and *SNHG3* has been observed; these are co-expressed with genes responsible for cell adhesion and biogenesis of cellular components. Of particular interest is *MIR22HG*, due to its known role in osteogenic differentiation of BM-MSCs [221]. Changes in genes associated with replication have also been reported. Specifically, compression leads to decreased expression of *Minichromosome Maintenance Complex Component 2* (*MCM2*), involved in prereplication complex assembly, as well as Proliferating Cell Nuclear Antigen (PCNA), a cofactor for DNA polymerase δ. These data indicate a reduction in replication activity under compression. Similarly, Cyclin A1 expression decreases, suggesting that mechanical forces influence the cell cycle [215]. Changes related to mitochondrial autophagy (mitophagy) are also noteworthy. For example, after mechanical stimulation, the ratio of LC3β-II to LC3β-I increases by approximately 1.67 times—a marker of autophagy induction. Levels of autophagy-related proteins Beclin1 and LAMP1 also increase by 1.22 and 1.58 times, respectively [17]. Additionally, alterations in the expression of matrix metalloproteinases (MMPs) and their inhibitors under stretch conditions have been observed. For instance, at 10% stretch, MMP-8 secretion increases significantly from 3.2 to 38.8 pg/mL; at lower stretch levels, no increase is detected. Meanwhile, the production of the tissue inhibitor of metalloproteinases-1 (TIMP-1) begins to rise at 5% stretch (to 54.5 ng/mL) and reaches 71.2 ng/mL at 10%, with the highest TIMP-1/MMP-8 ratio observed at 5% stretch [19].

It is logical to consider shear stress as a distinct category of mechanical impact on cells. In contrast to the normal forces of tension (pulling perpendicularly) and compression (pushing perpendicularly), which primarily engage integrins, focal adhesions, and the cytoskeleton, shear stress is classified differently because it represents a tangential force parallel to the cell surface and engages fundamentally different mechanosensory mechanisms (e.g., primary cilia, glycocalyx, and specific ion channels like PIEZO) [234]. It has been described that, under this type of mechanical stress, active changes are observed in the proliferative potential of cells [216,235], as well as in F-actin density [235], and the expression of various immunoregulatory factors [236] and several adhesion proteins [216]. It has been noted that, when waveform microfibers are subjected to shear stress for 1 and 4 h, there are changes in the expression of *cyclin D* (*cycD*), which is a regulator of the cell cycle at the G1/S checkpoint, facilitating the transition of cells from the presynthetic to the synthetic phase, during which DNA replication occurs [216]. Furthermore, it has been shown that shear stress influences cellular proliferative activity, with the intensity of exposure playing a crucial role in this process. For example, quantitative assessment of EdU (5-ethynyl-2′-deoxyuridine)-positive cells demonstrated that this type of mechanical stress stimulates proliferation of PDLSCs at low to moderate levels of shear stress, while higher levels lead to a decrease in proliferative activity. Shi Q. and colleagues found that shear stress ranging from 1 to 6 dyn/cm^2^ enhances cell proliferation, while an intensity of 9 dyn/cm^2^ suppresses it. The maximum proliferative response was observed at approximately 6 dyn/cm^2^, increasing from about 24.41%  ±  4.01% to 43.13%  ±  6.03%. According to studies, cell proliferative activity under shear stress is associated with the protein Yap: inhibition of Yap results in a significant reduction in proliferation [235]. Moreover, this type of mechanical stimulation leads to an increase in E-cadherin expression, which in turn is a mediator of intercellular adhesion, responsible for cell interactions with the extracellular matrix, and functions as a transmembrane mechanotransducer [221,237,238]. Another protein whose expression increases upon applying shear stress to cells is periostin [216]. Periostin is a protein that can act as an adhesive molecule connecting cells with collagen fibers, thereby providing mechanical strength to the periodontal complex. Additionally, it has been demonstrated that periostin interacts via its EMI domain with collagen I, fibronectin, and Notch1, and through its Fas-1 domain with tenascin-C and Bone Morphogenetic Protein 1 (BMP-1). It has been described that these processes may involve signaling pathways such as PI3K/Akt/mTOR and αvβ3 integrin/extracellular-related kinase [237].

Studies have also shown that this type of mechanical stress results in an increased density of F-actin within cells. The MAPK signaling pathway, which includes the p38 protein, regulates this process. The expression of p38, in turn, increases under shear stress [235]. It is also known that p38 is involved in the regulation of Yap localization [235,239]. Moreover, according to some data, Yap activity depends on its localization, which correlates with the duration of mechanical stimulation. For example, it has been shown that short-term exposure (10 min) results in pronounced nuclear transport of Yap, whereas exposure from 30 min to 4 h leads to cytoplasmic translocation of Yap (nuc/cyt Yap). Regardless of localization, mechanical stimulation lasting from 30 min to 4 h activates connective tissue growth factor (CTGF) and ankyrin repeat domain 1 (ANKRD1)—downstream components of the Yap-associated signaling pathway. Additionally, it has been demonstrated that upstream kinases LATS1/2 in the Yap–Hippo signaling pathway alter their expression under shear stress. These kinases phosphorylate Yap, promoting its translocation into the nucleus. Specifically, at a shear stress of 6 dyn/cm^2^, the expression of pLATS1 decreases, indicating regulation of Yap activity via LATS1/2 under shear stress conditions. Thus, this type of mechanical impact promotes inhibition of LATS kinase phosphorylation, which in turn affects YAP activity and cell proliferation. It is also noteworthy that the aforementioned p38 protein inhibits pLATS expression and activates pAKT, which is responsible for F-actin polymerization [235]. In addition to the aforementioned effects of mechanical impact, shear stress also induces changes in the expression of various immunological factors and cytokines. For example, it has been shown that exposure to a shear stress of 5 dyn/cm^2^ for 3 h results in increased expression of indoleamine 2,3-dioxygenase (IDO) and COX2 [236]. IDO is involved in the degradation of the essential amino acid tryptophan via the kynurenine pathway. This factor participates in the regulation of T-cell function [240]. Changes in the expression of genes responsible for inflammation are also characteristic of PDLSCs subjected to this type of mechanical stimulation. For instance, an increase in PTGS2 and CXCL8/IL8 activity has been observed [241].

Analysis of conditioned media from cells exposed to shear stress revealed increased secretion of transforming growth factor-beta 1 (TGF-β1) at an intensity of 5 dyn/cm^2^, IDO at 5 and 10 dyn/cm^2^, and kynurenine at 5 dyn/cm^2^. It was demonstrated that this process involves the ERK1/2 signaling pathway. Furthermore, the literature reports indicate that shear stress promotes the secretion of TGF-β1 and kynurenine, which in turn inhibit proliferative activity of T-cells and promote their differentiation into regulatory T cells (Treg) [236]. Additionally, it is known that shear stress leads to changes in *FOS* gene expression at an intensity of 6 dyn/cm^2^ for 1 h [241]. Interestingly, the *FOS* gene encodes a subunit of the AP-1 transcription factor dimer (activator protein-1), which regulates processes such as osteoblast proliferation and differentiation [242]. At similar levels of intensity and duration of exposure, increases in *RUNX2* and *VEGFA* expression are also observed; however, no changes are seen in *Osterix* and *OPG* expression [241]. It is also important to mention that RUNX2 is considered one of the key factors responsible for the osteogenic differentiation of MSCs. This process is thought to involve ERK1/2 and MAPK pathways [243].

**Figure 3:**
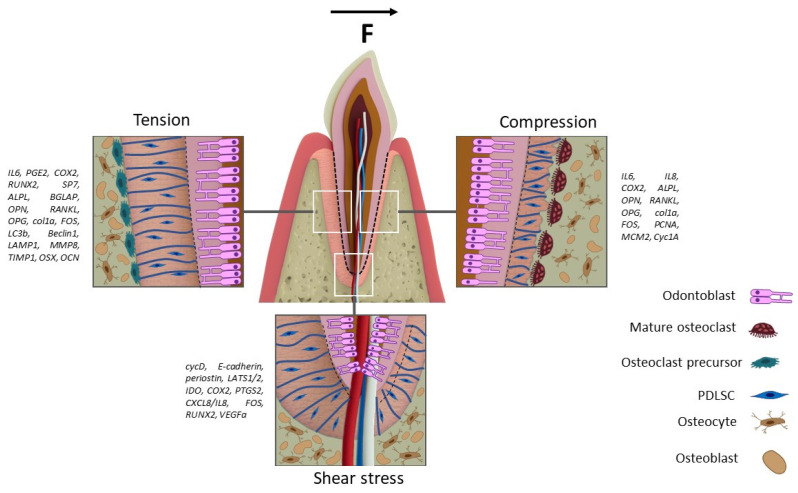
Mechanical stimuli on periodontal complex.

The effects of mechanical stimuli on ECM are in Table 2. The effects of mechanical stimuli of gene expression, protein synthesis, and the secretion of specific cytokines and microRNAs are summarized in Table 3. Figure 3 shows the key genes whose expression is altered under mechanical stress.

Periodontal tissue requires various types of mechanical stress to function properly. Alterations in gene expression across diverse functional categories are typical cellular responses to mechanical stimuli, underscoring the necessity for additional research in this area. As previously indicated, multiple studies have demonstrated the diverse reactions of PDLSCs to mechanical stimuli of varying intensities and durations, with these responses also showing variability across different types of mechanical forces and culture days. In summary, it seems that stretching is the most effective form of mechanical stimulation for initiating osteogenesis, as it activates pro-osteogenic genes [16]. Currently, the research in this field has not specified the optimal cultivation periods and stimulus intensities due to variations in experimental studies conducted by different authors. On the other hand, mechanical stimuli such as compression promote bone resorption by triggering osteoclastogenesis. Compression significantly contributes to the organization of fibers and other constituents of the extracellular matrix, which is essential for the formation of functional tissue [244]. The ideal balance between the intensity and duration of mechanical stimulation is expected to establish the most advantageous conditions for cellular proliferation and periodontal regeneration. The balance between pro-inflammatory and anti-inflammatory processes is also vital in the regeneration of the periodontal complex. Pro-inflammatory factors facilitate the activation of cells involved in tissue repair [245], while anti-inflammatory factors promote the transition of the inflammatory process into the recovery phase by stimulating collagen synthesis, new matrix formation, and the restoration of structural elements of the periodontium [246]. Thus, the obtained results indicate that, in the field of tissue engineering, researchers need to identify the desired cellular responses and modify parameters to achieve optimal cell behavior for periodontal regeneration.

**Table 2:** Effect of mechanical stimuli on ECM.

ECM Component	Stretching	Compression	References
Collagen I	↑ gene expression, protein ↓ fibers on immunohistochemistry	↓ gene expression, protein ↓ fibers on immunohistochemistry	[59,247,248,249]
Collagen III	↑ fibers on immunohistochemistry	↓ fibers on immunohistochemistry	[249]
Fibronectin (FN1)	↑ gene expression, protein	↓ gene expression	[59,250]
Osteopontin	↑ gene expression	NA	[250]
Osteonectin	↑ gene expression	NA	[250]

↑—increase in expression/quantity; ↓—decrease in expression/quantity; NA—not available.

**Table 3:** Effects of mechanical stimuli on cells.

Stimuli	Type of Cells	Description of the Bioreactor Design	Changes in Gene Expression	Changes in Protein Expression	Changes in Cytokine Production	Changes in microRNA Levels	Magnitude	Frequency	Duration	Medium	Ref.
Stretching	PDLSC	6-well plate BioFlex^®^ (Flexcell International Corporation, Burlington, VT, USA) with elastic membrane at the bottom; stretching achieved by a pushing mechanism from beneath the membrane.	*RUNX2* (↑1d → ↓3d; ↑3% → ↓20%); *ALPL* (↑1d → ↓3d; ↑3% → ↓20%); *Osterix* (↑1d → ↓3d; ↑3% → ↑↑6% → ↓20%); *OCN* (↓1d → ↑3d; ↓3% → ↑↑10% → ↑↑15% → ↑20%); *FOS* (↓1d →-↑3d; no dependence on the percentage of stretching.); *IL6* (↑1d → ↓3d; ↑3% → ↓10% → ↑20%); *PTGS2* (↑1d → ↓3d; ↓3% → ↑20%)	___	↑ IL1β ↑ TNFα ↑ IL6 ↑ IL8 ↑ PGE2	___	3%, 6%, 10%, 15%, 20%	NA	1, 2, 3 d	Low-glucose DMEM supplemented with 10% FBS, 2% MEM vitamins and 1% of antibiotic/antimycotic	[16]
	PDLSC (modification related to YAP)	Tension Plus System: 6-well plate with an elastic membrane at the bottom. Stretching was generated by pulling the membrane inward using a vacuum pump.	After 3 days of stretching in osteogenic differentiation medium: ↑↑ *OPN* ↑ *RUNX2* ↑ *Col1* ↑ *ALPL* ↑ *Osterix* ↑ *OCN*	↑ OPN ↑ OCN ↑ ALP	___	___	10%	0.1 Hz (5 s stress and 5 s rest)	24 h (mRNA extraction); 72 h (ALP staining, Western blots analyses and immunofluorescence detections)	Osteogenic differentiation media (α-MEM containing 10 mM β-glycerophosphate, 0.5 μM dexamethasone, 50 mg/mL ascorbic acid, and 10% FBS)	[217]
	PDLSC	6-well plate BioFlex combined with the Flexercell FX4000 Strain Unit. Stretching was generated by pulling the membrane inward using a vacuum pump.	↑ *OCN* ↑ *ALPL*	___	___	↓ miR-434-5p ↓ miR-1297 ↓ miR-3607-5p ↓ miR-145-5p ↓ miR-4328 ↓ miR-224-5p ↓ miR-195-5p	12%	6 cycles/min (5 s on and 5 s off)	6, 12, 24, 48, 72 h	αMEM supplemented with 10% FBS, 100 U ml−1 penicillin, and 100 mg ml−1 streptomycin	[228]
	PDLSC	Elastic membrane with a circularly fixed membrane, which is subjected to hydrostatic pressure and/or using a plate with Teflon rings.	↑ *Col1A1*	↑ Total Protein ↑ Col1 (10%, 24 h)	___	___	10%	30 cycles/min	24 h	Serum-free growth media containing 50 μg/mL of both ascorbic acid and β-aminopropionitrile	[59]
	PDLSC	Flexercell Strain Unit (Flexcell Corp).	___	___	↑ IL1β (3, 5 days)	___	9%, 18%	6 cycles/min (5 s elongation and 5 s relaxation)	1, 3, 5 d	αMEM supplemented with 100 μg/mL penicillin-G, 50 μg/mL gentamycin sulfate, 0,3 μg/mL amphotericin B, 2% fetal calf serum (FCS)	[58]
	PDLSC	BioFlex c гибким днoм (Flexcell), Flexcell^®^ FX-6000 TM Tension Unit (Flexcell International Corporation, Burlington, VT, USA).	↑ *OPN* (12, 48 h); ↑↑ *OPN* (24 h); ↑ *RUNX2* (12, 24 h); ↑ *OCN* (12, 24, 48 h); ↑ corresponding *LC3B-II/LC3B-I*; ↑ *Beclin1*; ↑ *LAMP1*.	↑ OPN (12, 24, 48 h); ↑ RUNX2 (12, 24 h); ↓ RUNX2 (48 h); ↑ OCN (12, 24, 48 h); ↑ corresponding LC3B-II/LC3B-I; ↑ Beclin1; ↑ LAMP1.	___	___	12%	0.1 Hz 6 cycles/min (5 s on and 5 s off)	12, 24, 48 h	α-MEM, 100 U/mL penicillin/streptomycin and 10% FBS	[17]
	PDLSC	Collagen-coated 6-well BioFlex^®^ (Flexcell International Corporation, Burlington, VT, USA)culture plates + Flexcell^®^ tension system (Flexcell International Corporation, Burlington, VT, USA)	↓ *FGFR2* (72 h); *↓ NOG* (72 h); ↓ *SULF1* (72 h); ↓ *SFRP1* (72 h)	___	___	___	Starting with a maximum tension intensity of 10% for the first 6 h and then gradually decreasing to 3%	NA	72 h	αMEM supplemented with 10% fetal calf serum (FCS), 2 mM L-glutamine, 100 U/mL penicillin, 100 μg/mL streptomycin and 2.5 μg/mL amphotericin	[229]
	PDLSC	6-well BioFlex^®^ plates with flexible membranes; (Flexcell International Corporation, Burlington, VT, USA)coated with fibronectin + Flexercell Strain Unit (Model FX 3000) (Flexcell International Corporation, Burlington, VT, USA).	↑ *IL-6* (10%, 12 h) ↓ *IL-6* (1, 5%, 12 h) ↑ *COX-2* (10%, 12 h)	↑ PGE2 10%, 12 h) ↑ IL-6 (10%, 12 h) ↑ MMP-8 (10%, 12 h) ↑ TIMP-1 (5, 10%, 12 h) ↑ TIMP-1/MMP-8 (5%, 12 h)	___	___	1%, 5%, 10%	NA	12 h	DMEM containing 1% l-glutamine, and 1% penicillin/streptomycin/neomycin	[19]
	PDLSC	6-well BioFlex^®^ plate (Flexcell International Corporation, Burlington, VT, USA) combined with the Flexercell^®^ 5000 unit.	↑ *RUNX2* (12 h); ↑ *Col1A1* (12 h); ↑ *CYTOR* (12 h); ↑ *MIR22HG* (12 h); ↑ *SNHG3* (12 h); ↑ *EGFR* (12 h); ↑ *FGF5* (12 h); ↑ *HIF1A* (12 h); ↑ *VEGFA* (12 h); ↓ *FOXO1*(12 h)	↑ RUNX2 (3 d.); ↑ Col1A1 (3 d.)	___	___	12%	NA	12 h, 3 d	αMEM supplemented with 100 U/mL penicillin, and 0.1 mg/mL streptomycin	[221]
Compression	PDLSC	Elastic membrane with a circularly fixed membrane, which is subjected to force using a plate with Teflon rings.	↓ *Col1A1*	↓ Col1 ↓ FN	___	___	10%	30 cycles/min	24 h	Serum-free growth media containing 50 μg/mL of both ascorbic acid and β-aminopropionitrile	[59]
	PDLSC	6-well plate + application of force using round glass cylinders.	↓ *PCNA* (24, 48, 72 h); ↓ *MCM2* (24, 48, 72 h); ↓ *Cyclin A1* (24, 48, 72 h);	↓ PCNA (24, 48, 72 h); ↓ MCM2 (24, 48, 72 h); ↓ Cyclin A1 (24, 48, 72 h);	↑ IL6 ↑ IL8	___	2 g/cm^2^ (0.02 N/cm^2^, respectively)	NA	24, 48, 72 h	DMEM containing 100 units/mL of penicillin, 100 µg/mL of streptomycin, 10% FCS and 50 mg/L-ascorbic acid	[215]
	PDLSC	Three-dimensional cell culture on PLGA scaffolds placed in a 6-well plate, with a cover glass and a granule bottle positioned on the scaffold with cells to generate a compression of 25 g/cm^2^.	↑ *RANKL* (6 h); ↑ *NFATC2* (6, 12 h); ↓ *OPG* (3 h); ↑ *OPG* (12 h); ↓ *OPG/RANKL* (3, 6 h); ↑ *OPG/RANKL* (12 h)	↑ RANKL (12 h); ↑ OPG (12 h)	___	___	25 g/cm^2^	NA	3, 6, 12 h	NA	[60]
	PDLSC	6-well plate + application of force using round glass cylinders (2 g/cm^2^).	↑ *VEGFA* (24 h);	↑ IL6 (24 h); ↑ TLR4 (3 h); ↓ TLR4 (24 h); ↓ pAKT (3, 24 h); ↑ pERK (3 h); ↑ p-p38 (3, 24 h);	↑ IL6 (24 h); ↑ IL8 (24 h); ↑ COX2 (24 h);	___	no information	NA	3, 24 h	NA	[219]
Shear stress	PDLSC	24 mm long collagen microfiber with PDLSC.	waveform microfiber: ↑ *CycD* (1, 4 h); ↑ *E-cadherin* (1, 4 h); ↑ *Periostin* (1, 4 h)	___	___	___	6 dyne/cm^2^	4.2 mL/min	1, 4 h	NA	[216]
	PDLSC	Parallel plate flow chamber.	↑ *CTGF* (30 min.–4 h); ↑ *ANKRD1* (30 min.–4 h); ↓ *pLATS1* (5 min.–4 h) ↑ p38	↓ Yap (30 min.-4 h);	___	___	1, 3, 6, and 9 dyn/cm^2^	NA	5, 10, 30 min, 1, 2, 4 h	High DMEM supplemented with 10% FBS and 0.1 mg/mL penicillin/streptomycin	[235]
	PDLSC	Cells into 35 mm culture dishes with a cone-shaped rotating disk.	↑ *IDO* (5 dyn/cm^2^, 3 h); ↑ *COX2* (5 dyn/cm^2^, 3 h);	↑ TGF-*β*1 (5 dyn/cm^2^); ↑ kynurenine (5 dyn/cm^2^); ↑ IDO (5 и 10 dyn/cm);	___	___	0.5, 5 and 10 dyn/cm^2^	NA	24 h	DMEM containing 10% FBS, 1% L-glutamine, 1% antibiotic–antimycotic	[236]
	PDLSC	A parallel flow chamber from polydimethylsiloxane (PDMS).	↑ *FOS* (6 dyn/cm^2^, 1 h); ↑ *PTGS2* (6 dyn/cm^2^, 1 h); ↑ *CXCL8/IL8* (6 dyn/cm^2^, 1 h); ↑ *RUNX2* (6 dyn/cm^2^, 1 h); ↑ *VEGFA* (6 dyn/cm^2^, 1 h);	___	___	___	1, 6 dyn/cm^2^	NA	1 h	DMEM/F-12 supplemented with 10% FBS, 1% MEM vitamins, 1% GlutaMAX™, 1% antibiotic/antimycotic, HEPES, odium pyruvate solution	[241]

↑—increase in expression/quantity; ↓—decrease in expression/quantity; NA—not available.

## 7. Future Perspectives

Among the various types of stem cells described, each can potentially be effective for periodontal disease (PD) therapy. Periodontitis is a complex disease characterized by the degeneration of structures such as the gingiva, periodontal ligament, cementum, and AB [2]. The effectiveness of each cell source is influenced by its specific properties, and the choice of source ultimately depends on the specific requirements of the treatment and the underlying causes of the disease. Given that periodontitis is a chronic inflammatory condition, UC-MSCs or AT-MSCs can be particularly effective due to their anti-inflammatory properties. Although bone-marrow-derived MSCs are multipotent and can improve regeneration of all periodontal components, including PDL fibers, cementum, and AB, their harvesting is highly invasive and traumatic, making them unsuitable for clinical use [67,79,251].

Odontogenic cells, such as DPSCs, PDLSCs, and GSCs, are frequently categorized separately, although they share many characteristics with MSCs. DPSCs have demonstrated efficacy in periodontal treatment for over 20 years. They can be easily harvested from extracted human teeth and serve as precursors for odontoblasts, which are essential for dentin repair, thereby exhibiting high regenerative potential [44]. Recently, PDLSCs have gained recognition as an ideal cell source for periodontal tissue regeneration; however, obtaining these cells from healthy donors presents certain challenges. As a result, some researchers have focused on utilizing PDLSCs derived from inflamed periodontal tissues (iPDLSCs). While iPDLSc remain effective in treating periodontitis, they exhibit reduced osteogenic differentiation capacity and diminished immunosuppressive potential compared to their healthy counterparts [252,253]. Regarding GSCs, research findings have been inconsistent due to the high heterogeneity of these cells, leading to a decline in attention towards their application in periodontal therapy [67]. It has been suggested that a combination of different cell types could offer the best solution for stimulating regeneration in periodontal tissues [57]. However, this approach may also be more expensive and time-consuming. Consequently, selecting the most suitable cell type remains a significant challenge, as researchers must strive to find a balance between therapeutic efficacy, cost-effectiveness, and the invasiveness of the procedure.

Tissue engineering for periodontal regeneration is limited because the periodontal area has a multiphasic and multicellular composition; for instance, engineers must simultaneously consider both PDL and bone regeneration while also inhibiting gingival downgrowth [254]. This complexity makes engineering constructs for periodontal tissue regeneration more challenging. Recent designs of more sophisticated structures, such as multiphase scaffolds, show promise for enhancing the precise regeneration of each component; however, researchers must still maintain effective control over spatial regeneration within the tissue and carefully consider its cellular and biomaterial aspects [255]. Using multifunctional, versatile biomaterials and incorporating different cell types in sophisticated spatial patterns could improve the final area regeneration. Future developments will likely rely on intricate, multi-dimensional strategies that effectively integrate advanced biomaterials (such as gradient scaffolds and smart hydrogels), precise cell sources (stem cells, induced pluripotent cells, or co-cultures), and spatially regulated structural designs (replicating native tissue interfaces, alignment, etc.). To systematically design these intricate systems, computational modeling and simulation (such as finite element analysis and agent-based modeling) will be essential for forecasting biomechanical signals, nutrient diffusion, and cell–scaffold interactions [256]. Furthermore, machine learning (ML) and artificial intelligence (AI) can accelerate the discovery process by analyzing massive datasets from in vitro and in vivo studies, determining the best combinations of materials, growth factors, and structural parameters, and predicting patient-specific regenerative outcomes. Integrating these computational approaches into a “digital twin” framework for periodontal defects has the potential to transform regenerative design from an empirical trial-and-error approach into a predictive, personalized paradigm, ultimately improving clinical translation and efficacy [257,258].

Working with various types of mechanical stimuli has several limitations, especially when it comes to affecting specific cell types for the development of new approaches in regenerative medicine. For PDL tissue, applying mechanical forces is a physiological standard. However, mechanical stimuli that do not correspond to physiological levels can lead to disorder in the periodontal complex. The in vitro mechanical stimulation of PDLSCs, as discussed in this paper, yields varying results based on the type of mechanical force, its intensity, duration, and the specific days of culture. These results highlight the importance of selecting the appropriate parameters to apply force cells during mechanical exposure. Moreover, it is important to note that these values are only estimates, and determining the most physiologically relevant conditions for mechanical stimuli is a difficult task that places limitations on research activities. One of the main limiting factors in studying mechanical effects in vitro is correlating the force/intensity of these stimuli with physiological conditions in vivo [16]. For example, some authors suggest that horizontal tooth movement in the PDL tissue is most physiological at forces ranging from 8% to 25% [259]. Nevertheless, some other researchers tend to agree that, for instance, a 10% tensile force in vitro causes cells to experience mechanical stimuli similar to those in the body [16]. Another limitation concerns the choice of duration and intensity for which cells are exposed to mechanical forces. The length of stimulation can either approximate physiological norms or induce pathological responses. For example, when analyzing gene expression, it is challenging to choose time intervals that allow for the assessment of both early- and late-response genes [16]. As an example of intensity, evidence suggests that stretching at an intensity below 15% facilitates bone remodeling, whereas stretching at an intensity exceeding 20% markedly diminishes its efficacy [217]. To address this issue, studies can be conducted using various intensities and durations of mechanical stimuli to create a “map” of data illustrating how force magnitude influences the studied parameters [260]. Subsequently, these results can be compared with clinical manifestations under normal and pathological conditions. Another issue is ensuring uniform distribution of applied force [261]. Typically, when cells undergo stretching, compression, or shear stress in vitro, certain regions endure greater forces while others experience lesser forces. This variation leads to ambiguous results, especially when studying parameters such as gene expression changes under external forces [262]. To mitigate this problem, existing sources of mechanical stimulation (such as bioreactors) could be upgraded—this comprises a separate engineering challenge. Alternatively, it is possible to calculate the area subjected to—or other parameters subject to—approximately equal force intensity and analyze specific fragments accordingly [263]. In addition, when working with PDLSCs, additional constraints characteristic of primary cell cultures come into play: the need for regular cell isolation; heterogeneity within the obtained cell populations; patient-specific variability; limited cell quantities obtainable from a single donor; and phenotypic mobility during passaging, which restricts the number of passages used in experiments. To address patient-specificity issues, using cells from different donors may be considered a potential solution [264].

To summarize, achieving predictable and functional periodontal regeneration requires highly controlled, integrated strategies. This requires the careful selection of cell sources, sophisticated biomaterial design incorporating precise structural features, and the application of tailored mechanical stimulation protocols. However, coordinating the complicated relationship among these parameters—cell type, biomaterial properties, scaffold architecture, and the intensity, duration, and type of mechanical forces—is a significant challenge. To overcome this complexity and progress toward clinically viable bioengineered constructs, future efforts will need to use advanced computational tools. Machine learning, AI, and computational modeling provide powerful tools for analyzing large datasets, predicting optimal parameter combinations, and guiding the design process. The integration of these predictive tools with dynamic bioreactor systems will allow for precise, real-time control of the cellular microenvironment, including scaffold properties and mechanical cues. This synergistic approach, which combines intelligent computational design with controlled bioreactor conditioning, holds the promise of producing optimized, functional periodontal tissues in vitro, ready for successful clinical translation.

## Figures and Tables

**Table 1:** Stem cell effectiveness in periodontal tissue therapy.

Cell Type	Delivery Method	Animal	Animal Model	Results	Sample Size and Age	Ref.
BMMSCs	Cell suspension	Rat	Periodontitis caused by binding wire around the bilateral maxillary first molars and subsequently inoculating with *Porphyromonas gingivalis.*	Periodontal tissue regeneration (histology and X-rays). Inhibition of inflammatory mediators’ interleukin 1β (IL-1β), interferon-γ (IFN-γ), and tumor necrosis factor-α (TNF-α).	5 animals per group	[87]
hDPSCs	Cell sheets and cell suspension	Miniature pig	Periodontitis caused by surgical AB defect	Twelve weeks after transplantation. Both cell sheets and suspension caused bone repair (histology, CT, formation of cementum-like layer, b-globin expression). hDPSCs sheets demonstrated a greater ability for bone repair than hDPSCs suspension injection.	6 animals per group 12 months old	[43]
hDPSCs	Cells on Bio-Oss^®^ scaffold (Geistlich Pharma, Switzerland)	Dog	Periodontitis model caused by incision from the mandibular canine to the mid-buccal of the first premolar.	After 4 weeks, treatment with cells stimulated new cementum and PDL formation compared to the control group. No significant differences in amount of bone formation.	6 animals per group	[44]
hPDLSCs	Cell suspension	Mice	Periodontitis caused by removal of AB. Defect size 3.5 × 2 × 1.5 mm.	Analysis 2 or 4 weeks after cells injection. Cells increase the values of width of new bone, new bone area fraction and cementum-like tissue was observed. PDLSCs increase the abundance of non-pathogenic bacteria and inhibit growth of pathogenic bacteria.	-	[55]
hPDLSCs	hPDLSCs on decellularized amniotic membrane	Rat	Periodontitis model, caused by removing of the buccal bone, PDL, cementum, and dentin from the mesial root of the mandibular first molar to the second molar. Defect size 2 × 3 mm.	hPDLSCs on decellularized amniotic membrane significantly enhances formation of cementum, periodontal ligament, and bone (micro-CT and histological analysis).	22 7–8 weeks old	[56]
Rat PDLSCs and osteoblast-like cells (MC3T3-E1 cells)	Composite cell sheets	Mice	Periodontal tissue injury model caused by removing the PDL fibers and bone near the palatal surface of maxillary first molar tooth. Treatment after 8 weeks.	Complex cell sheet from PDLSCs and osteoblast-like cells promote the regeneration of PDL-like fibers and AB periodontal ligament structure in ectopic and orthotopic transplantation (azan staining for PDL-like fibers, IHC analysis for expression of periostin and OCN, micro-CT)	- 4–5 weeks old	[57]
hPDLSCs, human AB stem cells (hABSCs)	A calcium phosphate-coated melt electrospinning polycaprolactone (CaP-PCL) scaffold with cell sheets	Rat	Periodontitis model caused by removing AB and cementum covering the roots of the mandibular first molar,	Cell sheets from hPDLSCs and hABSCs stimulate periodontal attachment formation. At 4 weeks, mineralized tissue (micro-CT), new bone, cementum, and PDL were formed (IHC for bone sialoprotein and OPN, histology).	3 animals per group 12 weeks old	[66]
hABSCs		Rat				
Human gingival margin-derived cells (GMCs)		Rat		GMCs did not induce periodontal regeneration.		
PDLSCs	Biphasic electrospinning scaffold with autologous cell sheet	Sheep	Surgically created periodontal defects. Treatment with cell sheet on first day and healing analysis for 5 and 10 weeks	PDLSCs sheet therapy significantly increased BV/TV compared to control scaffold after 10 weeks of therapy.	5 animals per group	[67]
GSCs		Sheep		GC sheet therapy had no positive effect on periodontal tissues and even decreased BV/TV, cementum formation, and ligament fibers attachment compared to scaffold without cells.		
GSCs	Fibroin/chitosan oligosaccharide lactate hydrogel	Rat	Periodontitis caused by cotton ligatures were bilaterally ligatured at the subgingival portion of the maxillary second molars for 14 days for desired level of periodontal destruction.	Treatment with cells in hydrogel after 14 days of injury. Decreased alveolar bone loss (ABL) revealed by micro-CT after 2 and 8 weeks of treatment. Less inflammation, tissue destruction 2 weeks after cell treatment. Reduction in apical migration of epithelium after 8 weeks revealed by Masson staining. Hydrogel itself also reduced inflammation and destruction, but did not cause reduction in epithelium apical migration.	10 animals in each group -	[63]
STRO-1 positive GSCs	Collagen scaffold and deproteinized bovine cancellous bone	Miniature pig	Periodontal defects in the premolar/molar area.	Cell-loaded scaffolds led to higher CAL, PD, GR and radiographic defect volume between baseline and 12 weeks, and lower junctional epithelium length and connective tissue adhesion after 12 weeks.	8 animals 4 weeks old	[64]
GMCs	IL-1ra+ or IL-1ra- releasing HA-ECM	Miniature pig	Periodontal defects were induced in the premolar/molar area.	Treatment after 4 weeks post-periodontal defects formation with GMCs in HA with or without IL-1re significantly improved PD, CAL, and GR.		[64]
GMSCs	Cell sheets	Dog	Class III furcation defects (5 mm from the furcation fornix to the bottom of the defect) with addition of anaerobic bacteria into the defect.	Treatment enhanced new bone and cementum formation (*p* < 0.01). In the GMSCs group, newly formed Sharpey’s fibers anchored into the newly regenerated cementum and were arranged perpendicularly to the root surface in contrast to parallel newly formed fibers in the control group.	4 animals and 4 teeth from each	[65]
BM-MSCs	Biphasic electrospinning scaffold with autologous cell sheet	Sheep	Surgically created defect 6 mm × 5 mm size.	BM-MSCs sheet increase BV/TV compared to control scaffold after 10 weeks of therapy, but have no effect on cementum formation and ligament fibers attachment compared to scaffold without cells	5 animals per time point 4 defects for each animal	[67]
BM-MSCs	Cell suspension	Rat	Surgical periodontal fenestration defects (2 × 4 × 1 mm), analysis 15 and 30 days after treatment.	BM-MSCs caused higher bone and cementum formation, and OCN, OPN and OPG synthesis compared to control.	6 animals per group	[72]
Acetylsalicylic acid (ASA) treated BM-MSCs	Cell suspension	Rat	Periodontitis, caused by cotton ligatures with *Porphyromonas gingivalis.*	ASA-BM-MSCs treatment reduced inflammatory infiltration and AB loss, proved by IHC staining of OPG/RANK-L and micro-CT after 3 weeks.	6 animals per group 6 weeks old	[74]
AT-MSCs	Fibrin gel	Miniature pig	Periodontitis caused by furcation defects in mandibular premolars.	AT-MSCs caused new AB formation (micro-CT), collagen fiber formation (histology + hematoxylin and eosin (HE), Masson’s trichrome (MT) staining) and decreased neutrophil infiltration in periodontal defects.	8 animals 24–30 months	[76]
ADSCs AT-MSCs	b-TCP and a human cancellous freeze-dried graft (HCG)	Rat	Alveolar defect, 5 mm. Dissection of muscles and periosteum followed by maxillary bone and zygoma dissection.	AT-MSCs promoted the regenerative effect of b-TCP and human HCG for AB reconstruction. Eight weeks after surgery application of AT-MSCs with b-TCP and HCG, osteogenic genes expression (RUNX2, Osterix, alkaline phosphatase (ALP), and BMP-2) were stimulated. Micro-CT revealed BV and TT compared to autologous bone graft.	6 animals per group	[88]
ADSCs AT-MSCs	Cell suspension	Rat	Ligature-induced periodontitis. After 2 weeks, the ligature was removed, which caused gingival inflammation, ulceration, and creation of pocket.	The histological and scanning electron microscopy results revealed restoration of the AB level around mandibular first molar.	12 animals per group 6 months old	[10]
ADSCs AT-MSCs	Cell suspension	Mice	Bacteria (*Porphyromonas gingivalis*, *Fusobacterium nucleatum*, and *Prevotella intermedia*)-induced periodontitis in molar regions.	After 12 weeks of cementum, researchers observed regeneration, organization of PDL fibers, an increase in the number of PD vessels, and higher BMP-2 and OPN expression in the treated group.	6 animals per group 8 weeks old	[78]
ADSCs AT-MSCs	Cell suspension	Rat	Ligature-induced periodontitis (14 days)	Four weeks after the AT-MSCs treatment, healthy periodontal tissue was observed and the cells were perpendicular to the cementum and AB; multiple blood vessels; well-oriented periodontal cells and fibers; less inflammatory infiltration compared to control.	5 animals per group	[9]
UC-MSCs	β-tricalcium phosphate bioceramic (β-TCP)	Rat	Periodontitis, caused by removing 2.5 × 1.5 mm of the AB over the mandibular first molar roots and cementum covering the roots of the mandibular first molar.	Better (*p* < 0.05) formation of new bone tissues, cementum, and PDL fibers compared to β-TCP without cells at 8 weeks after surgery.	5 animals per group 4–6 weeks old	[89]
UC-MSCs	Bone collagen particles	Rabbit	Alveolar clefts obtained by removing the incisors on the left side of the upper jaw.	Bone collagen particles with UC-MSCs stimulated bone repair and regeneration and treated AB alveolar cleft lesions.	6 animals per group 1 or 2 months old	[84]

## Data Availability

No new data were created or analyzed in this study.

## Abbreviations

The following abbreviations are used in this manuscript:

AB	alveolar bone
ABC	alveolar bone cells
ADM	acellular dermal matrix
AT-MSCs	Adipose-tissue-derived mesenchymal stem cells
ALP	alkaline phosphatase
ALPL	gene encoding alkaline phosphatase
ASA	acetylsalicylic acid
bFGF	basic fibroblast growth factor
BGM	bioactive glass microspheres
BMP	bone morphogenetic protein
BMSCs	bone marrow stem cells
b-TCP	b-tricalcium phosphate
BV	tissue volume (bone volume)
BV/TV	bone volume/total volume
CAD	computer-aided design
CAL	clinical attachment level
CaP	calcium phosphate
COL	collagen
CT	computed tomography
CTGF	connective tissue growth factor
DBB	droplet-based bioprinting
dECM	decellularized extracellular matrix
dPDL	decellularized periodontal ligament
dTM	decellularized tooth matrix
DLP	digital light processing
DPSCs	dental pulp stem cells
DSCs	dental stem cells
DSP	dentin sialoprotein
DSPP	dentin sialophosphoprotein
ECM	extracellular matrix
EGFR	epidermal growth factor receptor
FDM	fused deposition modeling
FGF5	fibroblast growth factor 5
GelMa	gelatin methacryloyl
GMCs	gingival margin-derived cells
GR	gingival recession
GSCs	gingival stem cells
GTR	guided tissue regeneration
Gum–HA	gum-hydroxyapatite
HA	hyaluronic acid
HAp	hydroxyapatite
HE	hematoxylin and eosin
HGF	hepatocyte growth factor
HIF-1α	hypoxia-inducible factor 1-alpha
IDO	indoleamine 2,3-dioxygenase
IFN-γ	interferon-γ
IHC	Immunohistochemical
IL1β	interleukin 1β
IL6	interleukin 6
IL8	interleukin 8
MAPK	mitogen-activated protein kinase family
MCM2	minichromosome maintenance complex component 2
MMPs	matrix metalloproteinases
MyD88	myeloid differentiation primary response gene 88
NMMII	non-muscle myosin II (Rho/ROCK)
OCN	osteocalcin (bone gamma-carboxyglutamic acid-containing protein)
OPG	osteoprotegerin (TNFRSF11B)
OPN	osteopontin (SPP1)
PCL	polycaprolactone
PCNA	proliferating cell nuclear antigen
PD	periodontal desease
PDL	periodontal ligament
PDLSCs	periodontal ligament-derived stem cells
PGE2	prostaglandin E2
PLGA	poly(lactic-co-glycolic acid)
PTGS2	prostaglandin–endoperoxide synthase 2
RANK	kappa-B nuclear factor activator receptor
RANKL	receptor activator of nuclear factor kappa ligand
SA	sodium alginate
SCAP	stem cells from the apical papilla
SHED	stem cells from human exfoliated deciduous teeth
SLA	stereolithography
SOD	superoxide dismutase
TGF-β	transforming growth factor beta
TIMP-1	tissue inhibitor of metalloproteinases-1
TLR4	toll-like receptor 4
TLS	triple-layered structure
TNFα	tumor necrosis factor alpha
TRAF6	TNF receptor-associated Factor 6
TT	trabecular thickness of bone
UC-MSCs	umbilical cord-derived mesenchymal stem cells
VEGF	vascular endothelial growth factor
YAP	Yes-associated protein
